# Comparative physiological and proteomic analysis indicates lower shock response to drought stress conditions in a self-pollinating perennial ryegrass

**DOI:** 10.1371/journal.pone.0234317

**Published:** 2020-06-18

**Authors:** Fatemeh Raeisi Vanani, Leila Shabani, Mohammad R. Sabzalian, Fariba Dehghanian, Lisa Winner

**Affiliations:** 1 Department of Plant Science, Faculty of Science, Shahrekord University, Shahrekord, Iran; 2 Research Institute of Biotechnology, Shahrekord University, Shahrekord, Iran; 3 Department of Agronomy and Plant Breeding, College of Agriculture, Isfahan University of Technology, Isfahan, Iran; 4 Department of Cell and Molecular Biology & Microbiology, Faculty of Biological Science and Technology, University of Isfahan, Isfahan, Iran; 5 Core Facility Proteomics, Center for Biological Systems Analysis (ZBSA), University of Freiburg, Freiburg, Germany; Pacific Northwest National Laboratory, UNITED STATES

## Abstract

We investigated the physiological and proteomic changes in the leaves of three *Lolium perenne* genotypes, one Iranian putative self-pollinating genotype named S10 and two commercial genotypes of Vigor and Speedy, subjected to drought stress conditions. The results of this study indeed showed higher RWC (relative water content), SDW (shoot dry weight), proline, ABA (abscisic acid), nitrogen and amino acid contents, and antioxidant enzymes activities of S10 under drought stress in comparison with the two other genotypes. A total of 915 proteins were identified using liquid chromatography-mass spectrometry (LC/MS) analysis, and the number of differentially abundant proteins between normal and stress conditions was 467, 456, and 99 in Vigor, Speedy, and S10, respectively. Proteins involved in carbon and energy metabolism, photosynthesis, TCA cycle, redox, and transport categories were up-regulated in the two commercial genotypes. We also found that some protein inductions, including those involved in amino acid and ABA metabolisms, aquaporin, HSPs, photorespiration, and increases in the abundance of antioxidant enzymes, are essential responses of the two commercial genotypes to drought stress. In contrast, we observed only slight changes in the protein profile of the S10 genotype under drought stress. Higher homozygosity due to self-pollination in the genetic background of the S10 genotype may have led to a lower variation in response to drought stress conditions.

## 1 Introduction

Cool-season grasses, emerging from and adapted to cool climatic conditions are economically and ecologically among the most important species due to their critical role in CO_2_ assimilation as well as forage production for livestock farming utilization [1]. Perennial ryegrass (*Lolium perenne* L.) is the most frequently cultivated species considered properly for farming [2] as it combines high growth rates and supplies the nutritive value of fodder. However, Norris [3] and Turgeon [4] mentioned that perennial ryegrass is a drought-susceptible grass species, although there seems to be a noticeable genotypic variation for drought stress responses.

Drought stress is the main abiotic element limiting plant growth and crop output throughout the world [5], which is roughly limiting plant production in 25% of the world's land. Due to its geographical location, Iran has an arid (65%) to semi-arid (25%) climate condition and is generally considered a dry country. Therefore, drought stress is the most critical and common environmental stress that limits agricultural production in the country and reduces the efficiency of using semi-arid and rain-fed areas. In cool-season grass species, drought stress adversely affects leaf water potential, photosynthetic and carbohydrate accumulation, respiration, production of reactive oxygen species, lipid peroxidation, and denaturation of proteins (reviewed by Loka et al. [6]). However, many cool-season grasses can resist moderate drought and maintain their aerial growth to avoid and tolerate leaf dehydration. Also, they may be equipped with the strategies facilitating their survival of severe drought, mainly associated with dehydration avoidance and tolerance, which primarily occurs in meristematic tissues [7]. In some species and genotypes of grasses, summer dormancy is also another strategy that confers efficient survival of meristematic tissues through dehydration avoidance and tolerance [8].

The grass family (Poaceae) exhibits different reproductive strategies among more than 10,000 species, some promoting self-pollination, and others cross-pollination, due to mechanisms such as self-incompatibility and being dioecious [9]. *Lolium perenne* is commonly considered as an out-crossing species [10]. This breeding system maintains a high genetic variation within the progenies and keeps the plasticity of the variety against environmental instabilities [11, 12]. However, this will also prohibit the repeatability of results in scientific studies since keeping the background of genetic material is impossible. Recently, Torkian et al. [13] reported that some of the Iranian perennial ryegrasses have appropriate reproductive characters of seed production as well as turf characteristics, which are also self-pollinating. Therefore, they may be considered as a new source of germplasm for future turfgrass breeding programs. Before this, it needs to confirm the potential of this newly emerged material, especially their performance in drought stress environments.

Under drought stress conditions, numerous alterations are induced in plants' physiological and molecular characteristics. Physiological characters of plants, particularly those related to plant water status, have a great deal of importance in the growth and development of plants during water-stress conditions. In the present study, relative water content (RWC) has been used as an example of the characters which determine drought-stress tolerance of *Lolium* genotypes. Biomass is a crucial parameter that influences plant growth; therefore, the accumulation and distribution of dry mass are significant considerations when investigating the effect of drought stress on plant growth. The role of glutathione in the antioxidative defense system also provides a rationale for its use as a stress marker. In general, an initial stress response is related to changes in the glutathione redox state. By this, acclimation is marked by increased glutathione concentrations, increase in related enzyme activities, and a more reduced redox state of glutathione.

Among phytohormones, abscisic acid is the most important one that confers abiotic stress tolerance in crop plants [14, 15]. In drought conditions, ABA content in plants increases considerably, inspiring stress-tolerance effects that help plants adapt and survive under these stressful situations [16]. The 'omics' approaches are often applied to reveal molecular changes due to drought stress and to investigate samples collected from different populations or upon different physiological states, aiming to find molecules that would differentiate between these classes of samples. These molecular studies in *L*. *perenne* for understanding some underlying molecular mechanisms in response to drought stress have been somewhat limited. In other species, recently proteome analyses have opened the way to the identification of proteins involved in drought stress tolerance in rice, *Oryza sativa* [17], barley, *Hordeum vulgare* [18, 19], *Stipa purpurea* [20], *Poa pratensis* [21] and wheat, *Triticum aestivum* [22]. The effort described here related to the comparative physiological and proteomic analysis was applied to investigate the molecular events–due to drought stress tolerance- in three perennial ryegrass genotypes including S10 as a newly introduced breeding line from Iran with self-pollinating reproductive habit with superior seed and turf characteristics compared to Speedy (Speedy Green) and Vigor as cross-pollinating commercial genotypes.

## 2 Materials and methods

### 2.1 Plant material and experimental design

*Lolium perenne* plant material of this experiment consisted of one well-performed genotype of S10, selected from the previous experiment [13] and two commercial populations of Vigor and Speedy Green or in short Speedy from Barenbrug (Barenbrug Co., the Netherland). Thus, three genotypes in this research were examined in a pot culture in a greenhouse according to a completely randomized design with three replications. Greenhouse conditions consisted of 16h/8h of light/dark photoperiod at a temperature range from 18.3 to 25.5°C. Ten tillers (with approximately equal sizes) of each genotype previously grown in a field were selected and transplanted into every 60 pots (size: 20*15 cm) filled with a 1:3 (v/v) mixture of sand and soil. The soil was sandy loam in texture with pH = 7.78 and Electrical Conductivity of 1.34 Ds.m^-1^. All three genotypes were then propagated for two months before being used in the experiment. Then, plants were regularly irrigated and grown in controlled greenhouse conditions for two weeks before starting the drought stress treatment. For this treatment, pots were divided into two groups: well-watered plants were irrigated every two days (control), and water-stressed plants. Drought stress was induced on plants by limiting irrigation to ensure water depletion to 20% of the field capacity of the soil using theta probe (AT Delta-T Devices SM300, Cambridge, England) before re-irrigation for 30 days. After seven days of the first period of drought stress treatment, leaves were cut off and stored at -70°C for later protein extraction. Biochemical measurements for the three genotypes of *Lolium* under drought stress were carried out on 1 g of fresh leaves. Antioxidant enzymes activities, ABA hormone and glutathione contents were measured one week after treatment; and proline, total N and amino acid contents, and shoot dry weight were calculated three weeks after the initial drought stress. At the end of the experiment, the harvested plants in pots were washed with distilled water, followed by surface drying with filter paper. For each plant, the leaves and roots were separated. Then leaves and roots were dried for 15 min at 105°C and then at 65°C to a constant weight in an oven. The dry weights were then determined.

### 2.2 Relative water content (RWC)

Leaf relative water contents (RWC) were calculated with the method of Barrs and Weatherley [23]. The relative leaf water content (RWC) was calculated as 100*(FW-DW)/(TW-DW), where FW = fresh weight, DW = dry weight, and TW = turgid weight. TW was measured after saturating the water content of leaf discs for 24 hours. DW was measured after desiccating leaf discs at 60°C to air-dry state.

### 2.3 Proline content

A 0.1 g sample of the fresh leaf was ground in liquid nitrogen from all the *Lolium* genotypes which were subjected to drought treatment. Leaf proline content was analyzed at the 3rd week post-stress treatment. The content of free proline in leaves was determined as described by Troll and Lindsley [24].

### 2.4 Estimation of total nitrogen

For estimation of total N, 1 g of fine-ground leaf dry samples was digested with sulfuric acid, and assays were carried out according to the Kjeldahl method [25].

### 2.5 Extraction and measurement of ABA

ABA was extracted according to the procedure developed by Kelen et al. [26] with some modifications. Leaf samples (1g) were used for extraction. Leaf tissue was ground with a mortar and a pestle in liquid nitrogen and homogenized in 10 cm^3^ extraction solution containing butylated hydroxy-toluene (0.25 g) and ascorbic acid (0.24 g) dissolved in 90% methanol and stirred overnight at 4°C. The extract was filtered through a Whatman filter, and methanol was evaporated under vacuum. Then, pH of the aqueous phase was adjusted to 8.5 with 0.1 M phosphate buffer and then partitioned with ethyl acetate three times. After removal of the ethyl acetate phase, pH of the aqueous phase was adjusted to 2.5 with 0.2 M HCl. The solution was partitioned with ethyl acetate three times and then passed through anhydrous sodium sulfate. After, the ethyl acetate phase was evaporated under vacuum, and the dry residue containing hormones was dissolved in 0.5 cm^3^ of HPLC grade methanol and stored in vials at 4°C.

An aliquot (20 mm^3^) of the filtered samples was injected into a Waters Symmetry-C18 chromatographic column (250 × 4.6 mm) (USA) with isocratic elution at a flow rate of 0.7 cm^3^ min^-1^ at 25°C using a mobile phase containing acetic acid solution (0.2%) and methanol (100%) (50:50 v/v). Detection of ABA was carried out at 265 nm by a UV detector (Waters 2487) with known concentrations of (±)-abscisic acid (Sigma A1049).

### 2.6 Antioxidant enzymes and glutathione assays

Activities of catalase (CAT), superoxide dismutase (SOD), ascorbate peroxidase (APX), glutathione reductase (GR) and glutathione S-transferase (GST) enzymes were detected in leaf tissue extracts spectrophotometrically as described in our previous work [27]. Glutathione (GSH) content of the leaves was measured according to May and Leaver [28].

### 2.7 Amino acids assay

Amino acids were extracted from one gram of dry leaves according to the procedure described by Lisiewska et al. [29]. The chromatographic separation of 13 amino acids was achieved by the ion exchange chromatography in an amino acid analyzer (ARACUS, Germany). The identification of amino acids was carried out based on the retention time at the chromatographic separation. All determinations were carried out in two replications for each sample.

### 2.8 Proteomic analysis

Approximately 100 mg of plant material (fresh weight) was ground in liquid nitrogen using a pre-cooled mortar and pestle. The dry powder was transferred to 1.5 cm^3^ of precipitation solution (10% TCA w/v, 0.07% DTT w/v), according to Yang et al. [20]. The extract was left to precipitate at -20°C overnight. Precipitated proteins were then pelleted at 16,000 ×g for 15 min at 4°C. The pellet was washed at least three times with acetone containing 0.07% (w/v) DTT at -20°C for 30 min, then, the mixture was vortexed and centrifuged at 16,000 ×g for 20 min, at 4°C. The pellet was recovered and air-dried at room temperature and dissolved in 1 cm^3^ of 1x SDS sample buffer [30]. The solutions were vigorously vortex-mixed and kept at room temperature for one h and centrifuged at 14,000 ×g for 10 min at 4°C. The supernatant was transformed into a new tube and stored at 4°C overnight and run on a 5% stacking gel (in an SDS PAGE system with an equal volume of stacking (5%) and resolving (10%) gel) according to Raorane et al. [30] (S1 Fig). The single bands formed in stacking gel, containing condensed and purified total soluble proteins, were stained with coomassie brilliant blue (G-250) as described by Neuhoff et al. [31] and used in the following steps.

#### 2.8.1 Mass spectrometry and data analysis

After gel staining using coomassie, the gel was cut in 10 slices. Next, gel digestion was performed using trypsin, and peptides were extracted and desalted. The protocol reported by Rappsilber et al. was used for peptide extraction [32]. Then, liquid chromatography-mass spectrometry (LC/MS) analysis was performed on an LTQ Orbitrap XL (Thermo Fisher Scientific, Bremen, Germany) coupled to an Eksigent 2D nano flow-HPLC equipped with in-house packed C18 columns of approximately 20 cm length (Reprosil-Pur 120 C-18-AQ, 3 μm, Ammerbuch, Germany) without a pre-column. Because of an unavailable database for the proteins of *L*. *perenne* and limited protein database of *Lolium* genus, *Brachypodium distachyon* protein database was selected for the identification of proteins in this study. The analysis of MS raw data was performed using MaxQuant (v. 1.5.3.30 [33]) against the UniProt *Brachypodium distachyon* protein sequence database (released June 20, 2018; 44,786 entries in total). A false discovery rate of 1% was applied for both protein lists and peptide-spectrum matches (on modified peptides, separately). Analysis of protein groups was performed with the Perseus software [34]. Normalized ratios were log_2_-transformed, and a mean log_2_ was calculated across all three replicates. Finally, proteins with a p-value below 0.05 and fold change higher or lower than +1.5 and -0.5, respectively, were considered. The differentially abundant proteins were used for COG (Cluster of Orthologous Groups of proteins) category and annotation.

#### 2.8.2 Bioinformatics analysis

The proteomic data visualization in schematic metabolism pathways, as well as functional classification of proteins, was performed by using the MapMan (Version 3.6.0RC1, downloaded from http://mapman.gabipd.org/ Web Site) software program.

### 2.9 Statistical analyses

Statistical analyses were carried out with three biological and two technical replicates for proteomic analyses. The results of protein abundance were statistically evaluated by student's t-test by using the Multi Experiment Viewer (MEW) software considering *P*<0.05 as the critical significance level. Relative water content, shoot dry weight, total nitrogen, proline and glutathione contents, antioxidant enzymes activities, and ABA content were analyzed using ANOVA by the SAS software [35]. Significant differences between means were defined by LSD post-hoc test at *P*<0.05. PCA (Principal Component Analysis) was conducted utilizing STATGRAPHICS Centurion ver. 17 software (Statpoint Technologies, Inc., Warrenton, Virginia, USA).

## 3 Results

### 3.1 Physiological characteristics of ryegrass genotypes under drought stress

The results of statistical analyses for RWC, shoot dry weight (SDW), proline, ABA, total N, and glutathione contents of the three genotypes of *Lolium perenne* are presented in Table 1. RWC content of the three genotypes decreased significantly under drought stress (P<0.05). However, the RWC content of the S10 genotype was much higher compared to the other genotypes under both stress and control conditions. Also, the SDW of the genotypes decreased significantly under drought stress (P<0.05). However, like RWC, SDW was significantly higher in S10 than that of the other two genotypes under both stress and control conditions. At stress conditions, dry weights of Vigor, Speedy, and S10 genotypes decreased by 9.7%, 12.5%, and 5.5%, respectively, compared to the control condition.

**Table 1:** Relative water content (RWC), shoot dry weight (SDW), proline content, ABA concentration, total Nitrogen, and glutathione content of three genotypes of *Lolium perenne* under drought stress. Values represent means of n = 3 replicates.

G	RWC (%)		SDW (g)		Proline (μmol/g fw)		ABA (μmol/g fw)		N%		Glu (μg/g fw)	
	C	T	C	T	C	T	C	T	C	T	C	T
**V**	75±1^b^	59.75±0.08^d^	12.55±0.32^c^	11.3±0.23^d^	1845.52±2.4^e^	2305.52±2.3^c^	76.59±4.2^e^	473.4±38.4^c^	3.05±0.04^b^	2.5±0.1^c^	2.35±0.3^d^	4.67±0.2^b^
**S**	64.33±4.04^c^	61.89±0.21^d^	11.18±0.2^d^	9.78±0.09^e^	1803.75±5.0^f^	2274.41±3.6^d^	110.4±5.6^e^	567.63±12.4^b^	3.04±0.01^b^	2.46±0.05^b^	2.7±0.03^c^	2.96±0.15^c^
**S10**	83.42±3.00^a^	79.90±0.3^a^	14.35±0.05^a^	13.5±0.3^b^	2561.48±10.6^b^	5436.93±3.5^a^	314.9±20.7^d^	742.2±50.5^a^	3.4±0.09^a^	3.14±0.14^b^	2.74±0.07^c^	5.01±0.07^a^

Mean values followed by the same letter are not significantly different (P < 0.05).

G = genotype, V = vigor genotype, S = speedy genotype, C = control condition, T = treatment condition (drought).

Proline content of the three genotypes increased under drought stress (P<0.05). However, proline accumulation in S10 genotype was much higher than Vigor and Speedy genotypes under both drought and control conditions. The ABA concentration in the genotypes also increased under drought stress (P<0.05) and, like proline, the concentration of this hormone in S10 was significantly higher than that in the two commercial genotypes under both drought and control conditions. In all genotypes, the content of leaf total N decreased under drought stress (P<0.05). In contrast, drought stress significantly increased glutathione content in Vigor and S10 genotypes.

**Fig 1:**
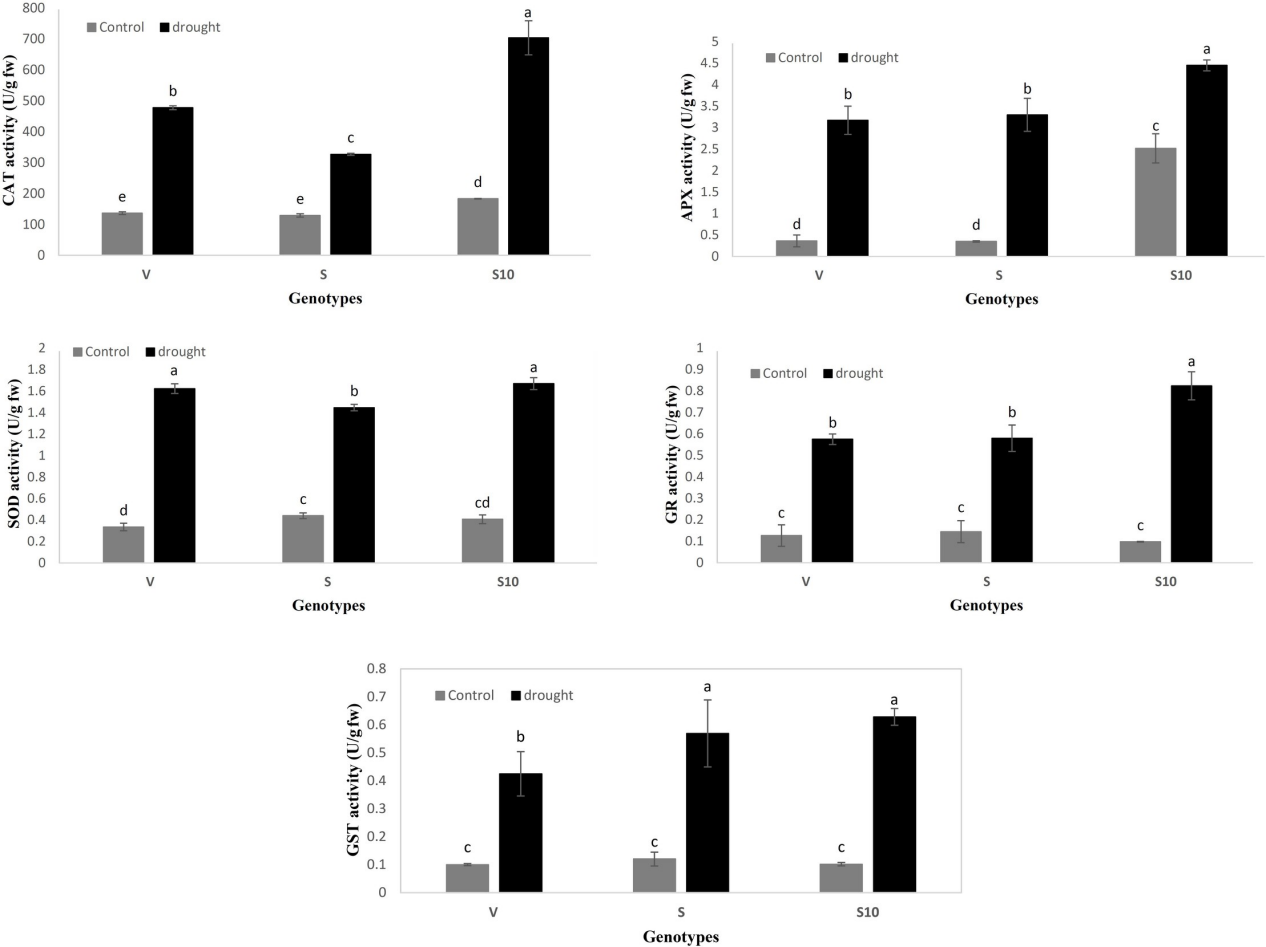
Antioxidant enzyme activities of three genotypes of *Lolium perenne* under drought stress. Bars represent standard error. Mean values followed by the same letter are not significantly different (p < 0.05). Different letters indicate significant differences by LSD test at *P* < 0.05, *n* = 3. V = vigor genotype, S = speedy genotype.

All of the tested antioxidant enzymes, including APX, SOD, CAT, GR, and GST, showed similar activity changes under drought stress in the three genotypes. However, the induction of APX activity in Vigor and Speedy was much higher than that of the S10 genotype under drought stress. In contrast, CAT and GR activities were higher in S10 genotype under drought stress conditions (Fig 1).

**Fig 2:**
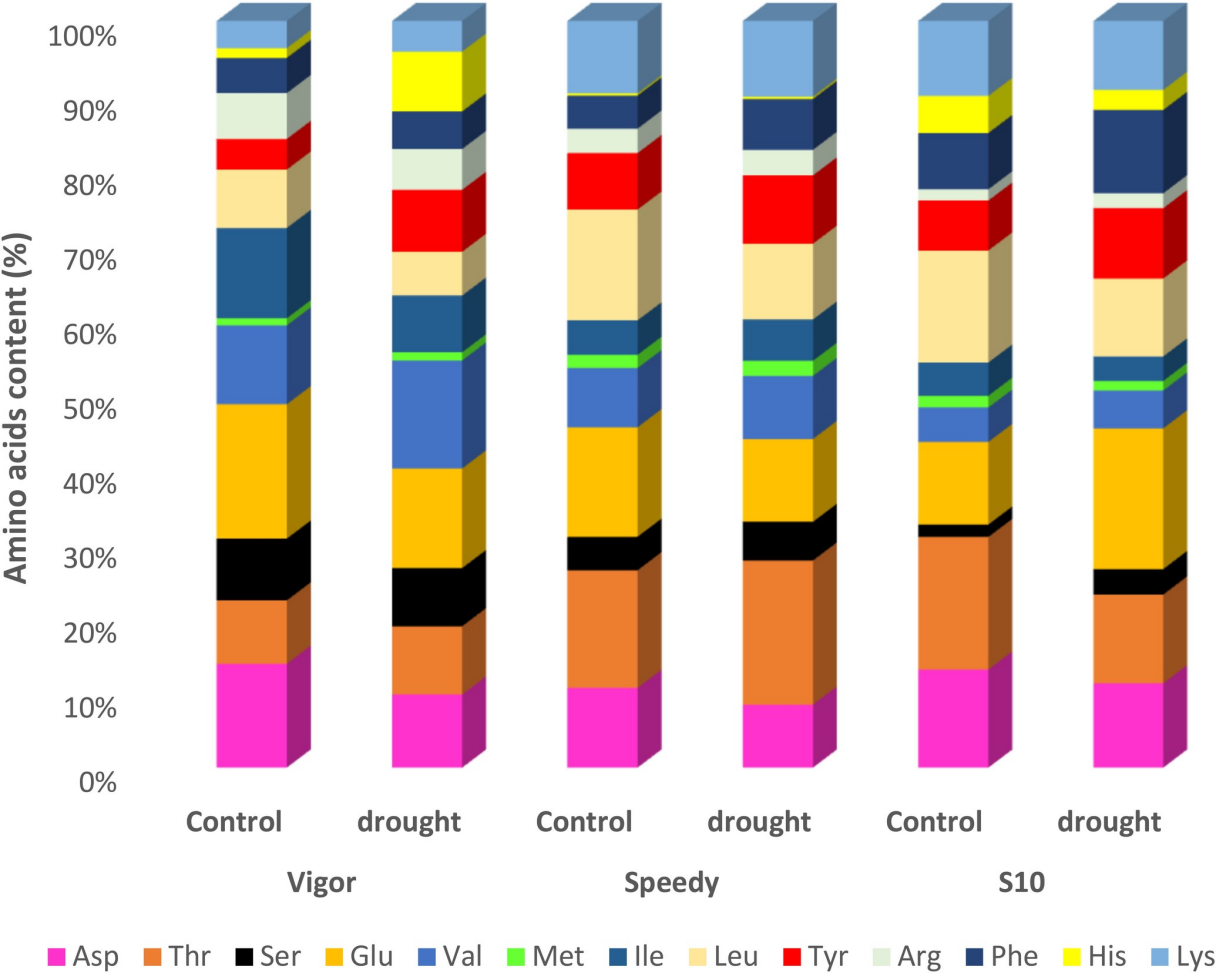
Amino acids content including Aspartic acid, Threonine, Serine, Glutamic acid, Valine, Methionine, Isoleucine, Leucine, Tyrosine, Arginine, Phenylalanine, Histidine and Lysine of three genotypes of *Lolium perenne* under drought stress.

The content of amino acids, including aspartic acid, threonine, serine, glutamic acid, valine, methionine, isoleucine, leucine, tyrosine, arginine, phenylalanine, histidine and lysine of the three genotypes was measured three weeks after drought stress (Fig 2). The results showed that the majority of the amino acid content of all genotypes increased under drought conditions. However, the total amino acid content of S10 was much higher than that of Vigor and Speedy under both control and drought stress conditions. Under stress conditions, the total amino acid content of S10 was 262 (mg/100 g DW) as against 219 and 217 (mg/100 g DW) in Vigor and Speedy genotypes, respectively.

### 3.2 Proteomic characteristics of ryegrass genotypes under drought stress

The small grass species, *Brachypodium distachyon*, is potentially an ideal model plant system for grass research. Therefore, the *Brachypodium* protein database was used for protein identification to dissect tolerance to drought stress in *Lolium perenne* genotypes. Based on the *Brachypodium* protein database, a total of 915 proteins were identified in each genotype. The number of proteins whose relative abundance level was significantly altered under drought stress conditions was 467, 456, and 97 in Vigor, Speedy, and S10 genotypes, respectively.

**Fig 3:**
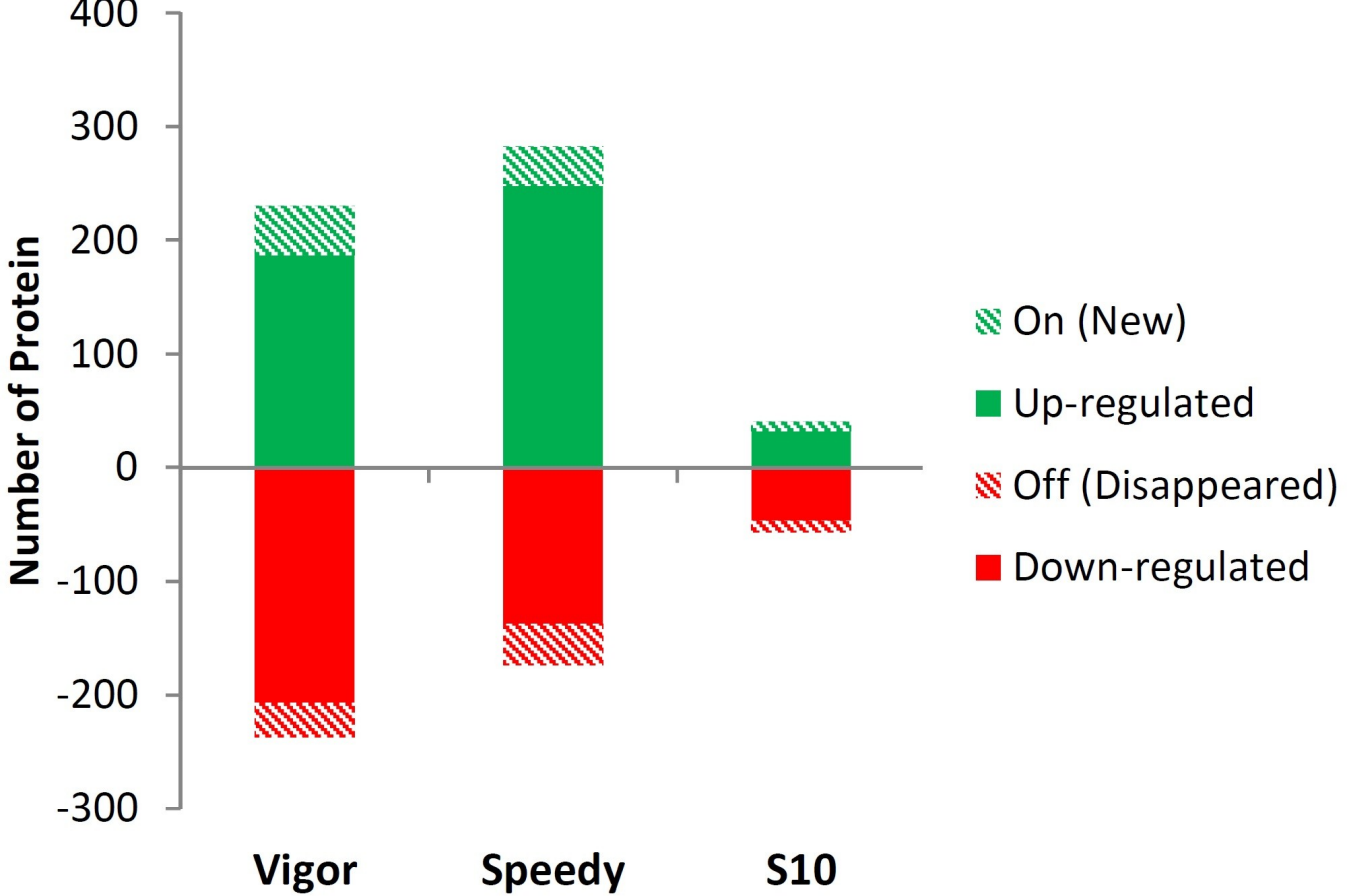
The number of up and down-regulated proteins in drought-stressed seedlings of Vigor, Speedy, and S10 compared to control plants of each genotype. “on (New)”: proteins in experimental plants that were not found in controls. “off (disappeared)”: Some proteins found in controls were absent in the experimental samples.

Both “up-regulated” and “on (New)” proteins are considered together as “differentially increased proteins." Similarly, the term “differentially decreased proteins” included both “down-regulated” and “off (disappeared)” proteins. Among the proteins, 230, 282 and 40 differentially increased proteins (fold change >1.5, P < 0.05) and 237, 174 and 57 differentially decreased proteins (fold change <0.5, P < 0.05) were found under drought stress compared to control condition in Vigor, Speedy, and S10 genotypes, respectively, (Fig 3, S1 Table).

### 3.3 Functional annotation of identified and differentially abundant proteins

**Fig 4:**
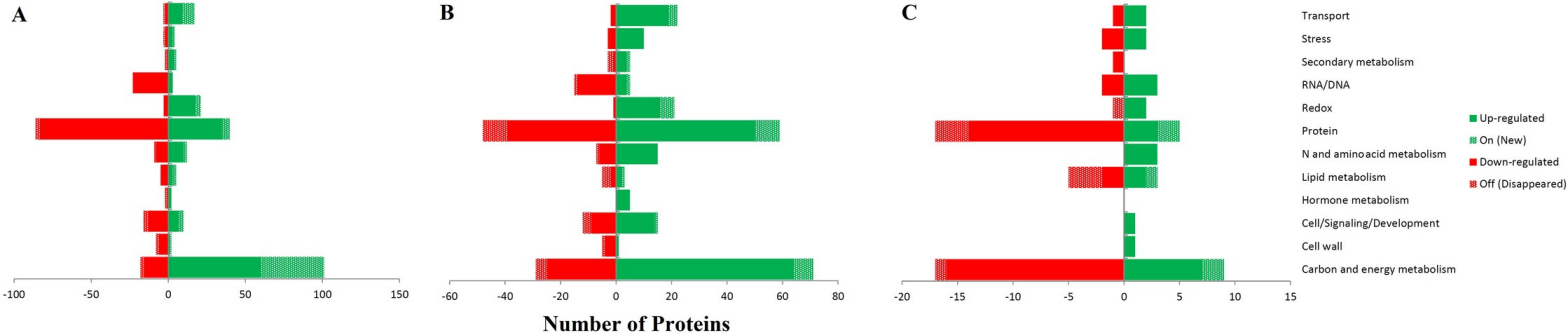
COG annotation of differentially abundant proteins of three genotypes of *Lolium perenne* (fold increase>1.5, fold decrease<0.5, *P* < 0.05). A: Vigor genotype, B: Speedy genotype, C: S10 genotype.

To simplify the analysis of protein expression, differentially abundant proteins were classified into 12 COG categories using Mapman ontology and the *Brachypodium* mapping file (Fig 4). Among 230 differentially increased proteins in the Vigor genotype, the most are involved in carbon and energy metabolism, protein, redox, and transport categories. Similarly, in Speedy genotype, among 282 differentially increased proteins, the most are involved in carbon and energy metabolism, protein, redox, stress, and transport categories. In contrast, in the S10 genotype, most of the proteins are involved in carbon and energy metabolism, and protein and stress categories decreased under drought stress (Fig 4).

Based on the COG annotation, drought stress induced changes in the abundance of proteins which are constituting the photosystem I (PSI) and PSII reaction centers and PSI and PSII polypeptide subunits. We found that proteins constituting reaction centers of the two photosystems (no. 6, 9, 17, 31, 54) increased in both Vigor and Speedy genotypes. In contrast, some proteins of reaction centers decreased in Vigor (no. 455) and S10 (no. 54) genotypes.

**Fig 5:**
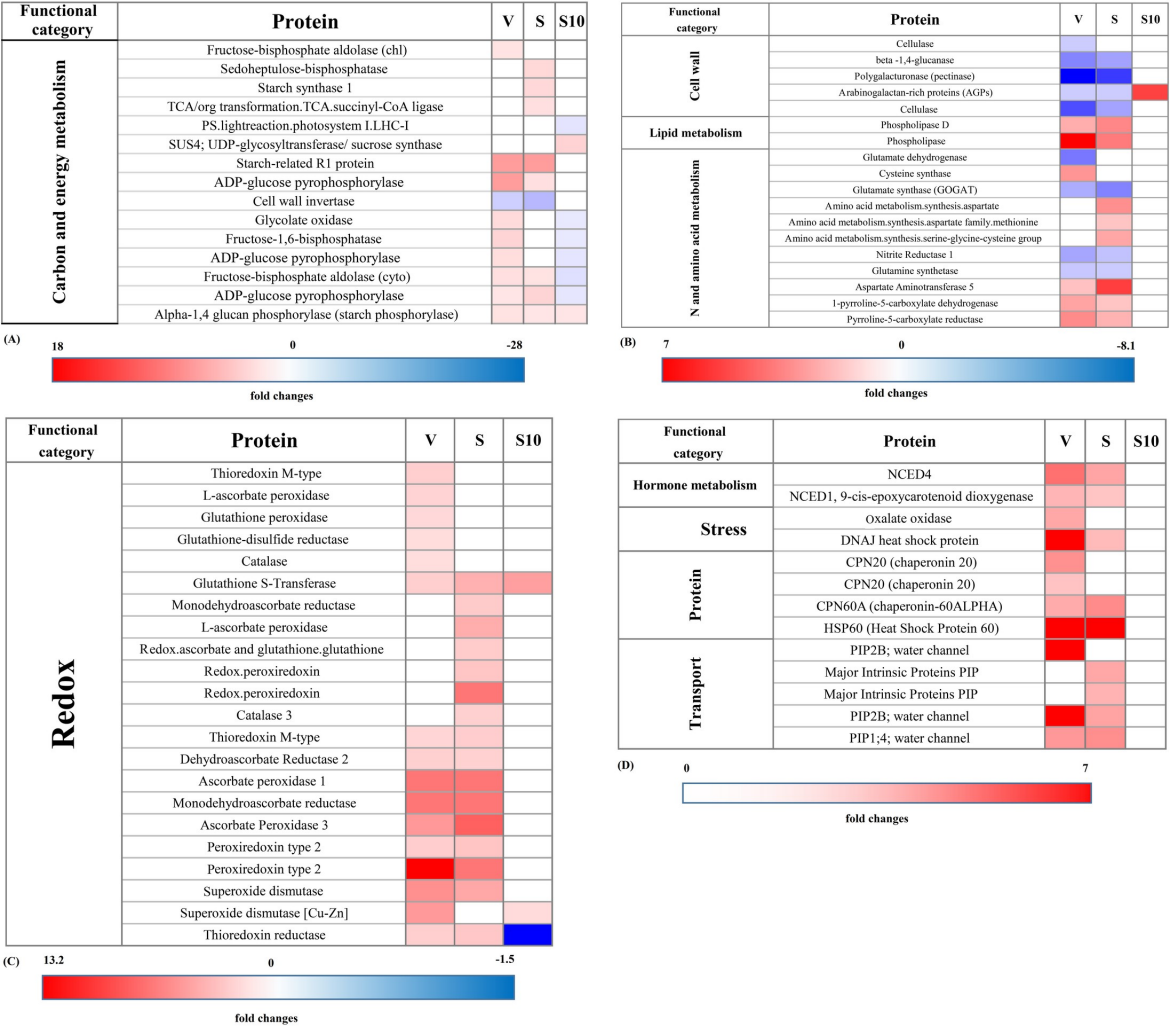
Drought-induced changes in the accumulation levels of selected proteins involved in carbon and energy metabolism category (A), cell wall, lipid metabolism, and N and amino acid metabolism categories (B), redox category (C) and hormone metabolism, stress, protein, and transport categories (D) identified in leaves of three genotypes of *Lolium perenne*. V = vigor genotype, S = speedy genotype.

Several polypeptide subunits of photosystems highly decreased in the three genotypes of *Lolium* under drought stress. We observed a drought-induced increase in the levels of small and large subunits of RuBisCO in Vigor and Speedy genotypes, whereas, in S10, such a change was not detected. In the Vigor genotype, RuBisCO activase exhibited drought-induced accumulation. The Expression level of the protein sedoheptulose-bisphosphatase, which participates in the Calvin cycle, was up-regulated under drought in Speedy genotype (Fig 5A, S1 Table). The chloroplastic isoform of fructose-1,6-bisphosphate aldolase (FBA) was down-regulated in Vigor and Speedy genotypes; however, no changes were detected in S10 genotype. In contrast, the cytoplasmic isoform of FBA was up-regulated in Vigor and Speedy genotypes, whereas it was down-regulated in the S10 genotype.

Alpha and beta subunits of pyruvate dehydrogenase complex, malate dehydrogenase, malic enzyme, citrate synthase, succinyl-CoA ligase, succinate dehydrogenase, isocitrate dehydrogenase, and aconitate hydratase, which are involved in the glycolysis pathway and Krebs cycle, increased in both Vigor and Speedy genotypes but did not change in the S10 genotype. However, the NADP dependent malic enzyme (NADP-ME) increased in all three genotypes.

Based on the COG annotation, the differentially abundant proteins involved in starch and sucrose metabolisms were up- or down-regulated in the three genotypes under drought stress. Regarding sucrose synthase-related proteins, we found that in S10 genotype, sucrose synthase (SS) increased, while fructose-1,6-bisphosphatase showed reduced levels (Fig 5A, S1 Table). We also found the up-regulation of starch-degradation enzymes, starch-related R1 protein, and starch phosphorylase in the three genotypes of *Lolium* in drought stress conditions (Table 1). On the contrary, the isoforms of ADP-glucose pyrophosphorylase involved in the pathway of starch synthesis were up-regulated only in Vigor and Speedy genotypes. In the S10 genotype, no changes were detected in some of the isoforms, and other isoforms showed down-regulated expression levels (Fig 5A, S1 Table).

Based on the COG annotation, the differentially abundant proteins in the cluster of cell walls, including cellulose, pectinase, β-xylosidase and β-1, 4-glucanase enzymes were down-regulated in Vigor and Speedy genotypes (Fig 5B, S1 Table). A significant increase in the protein abundance of arabinogalactan-rich proteins (AGPs) has also been found in the S10 genotype.

Our proteomic analysis revealed that drought-induced changes in concentration of proteins involved in lipid metabolism and two isoforms of the phospholipase enzyme increased in both commercial genotypes. Interestingly, opposite to these genotypes, proteins involved in lipid metabolism did not change or even were reduced in the S10 genotype under drought stress conditions (Fig 5B, S1 Table).

In the functional group of nitrogen and amino acid metabolism, some of the differentially abundant proteins increased in Vigor and Speedy genotypes and the proteins involved in the metabolism of methionine, cysteine, aspartate, glycine, and proline were up-regulated in these two commercial genotypes. Δ-1-pyrroline-5-carboxylate synthase (P5CS) enzyme, which catalyzes the first two steps in proline biosynthesis in plants, did not exhibit significant changes in the three genotypes of *Lolium*. However, drought-induced increase in the level of pyrroline-5-carboxylate reductase (P5CR), which is a part of the L-proline biosynthesis pathway, was significantly higher in Vigor and Speedy genotypes. Also, the abundance of 1-pyrroline-5-carboxylate dehydrogenase enzyme, which is a part of the pathway of L-proline degradation into L-glutamate, significantly increased in these two genotypes. We observed drought-induced reduction in the levels of nitrite reductase 1, glutamine synthetase, and glutamate dehydrogenase (involved in nitrogen flow) in Vigor and Speedy genotypes (Fig 5B, S1 Table).

Concerning the ROS-generation proteins, we found two differentially increased proteins, glycolate oxidase, and oxalate oxidase, in the two commercial genotypes. However, they decreased or did no change in drought-stressed S10 genotype. Under drought stress, plants respond to ROS-over abundance in cells by producing enzymatic and nonenzymatic ROS scavenging antioxidants. The increased level of dehydroascorbate reductase (DHAR), monodehydroascorbate reductase (MDHAR), catalase, ascorbate peroxidase (APX) and SOD was observed in leaves of both Vigor and Speedy genotypes. However, GR and glutathione peroxidase was up-regulated only in the Vigor genotype. Under drought stress, the up-regulation of glutathione S-transferase was found in the three genotypes of *Lolium*. The other hydrogen peroxide-decomposing enzymes, glutathione peroxidase, and peroxiredoxin and thioredoxin reductase increased only in Vigor and Speedy genotypes in response to drought stress (Fig 5C, S1 Table).

Given that ABA metabolism is a crucial aspect of the plant response to drought stress, proteins involved in ABA metabolism were searched in the regulated proteins. We found that two isoforms of 9-cis epoxy carotenoid dioxygenase (NCED1 and NCED4) increased during drought stress in Vigor and Speedy genotypes; however, the S10 genotype presented no alteration in this respect (Fig 5D, S1 Table).

Based on the COG annotation, the levels of four heat shock proteins (HSPs) (HSP60, chaperonin-60ALPHA, chaperonin 20 and DNAJ) increased under drought stress only in Vigor and Speedy genotypes; and no protein abundance changes of HSP-like proteins were detected in S10 genotype (Fig 5D, S1 Table).

Also, based on the COG annotation, some proteins of the transport system were up-regulated only in the two commercial genotypes. The PIP1;4 and PIP2B aquaporin proteins (AQPs) were up-regulated in Speedy and Vigor genotypes; however, the up-regulation level of these proteins was higher in Speedy genotype. Furthermore, the PIP2A protein and two isoforms of PIP were also up-regulated in the Speedy genotype (Fig 5D, S1 Table).

### 3.4 Multivariate data analysis

**Fig 6:**
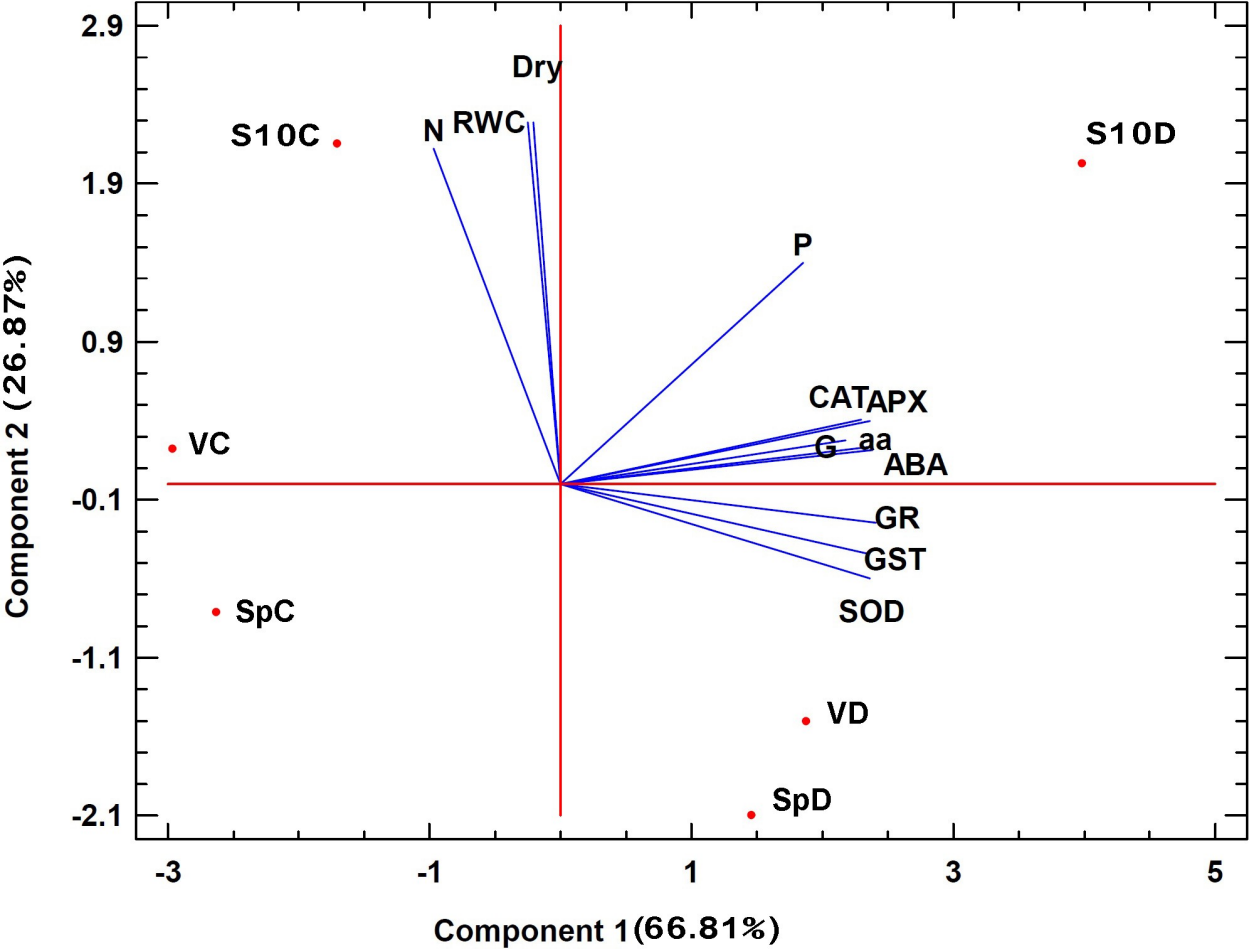
The two-dimensional biplot of PCA (principal component analysis) indicating the correlation between the three genotypes of ryegrass, drought stress, and all examined characteristics. **C**: control condition, **D**: drought condition, **V**: Vigor genotype, **Sp**: Speedy genotype, **S10**: S10 genotype, **CAT**: Catalase activity, **APX**: Ascorbate peroxidase activity, **SOD**: Superoxide dismutase activity, **GST**: Glutathione-S-transferase activity, **GR**: Glutathione reductase activity, **G**: Glutathione content, **ABA**: ABA concentration, **W**: RWC content, **Dry**: shoot dry weight, **P**: proline content, **N**: Nitrogen total, **aa**: total amino acids content.

Principal Component Analysis (PCA) was conducted to determine the correlation between the three genotypes of ryegrass, drought stress, and all the examined characteristics. Noteworthy, the PCA analysis displayed a high correlation between biomass, total N, and RWC content (Fig 6); however, no positive correlation between biomass and total N with amino acids total content and antioxidant enzymes was found. The separation of S10 genotype from the two commercial genotypes (Vigor and Speedy) can be easily deduced from the data provided. Also, we found that the S10 genotype in drought conditions had a combination of higher dry weight (second component) and antioxidant enzymes (first component) than those in the other two genotypes. Therefore, under drought, better biomass and antioxidant potential along with higher drought tolerance are undoubtedly ascribed to this genotype in comparison to the two commercial genotypes. The PCA results also revealed the separation of control and drought conditions for all tested genotypes and indicated that drought stress was particularly distinctive by induction in the production of antioxidant enzymes. This PCA analysis may suggest that the defense strategy utilized by the two commercial genotypes to assist plants against drought is different from the mechanism used by the well-performed S10 genotype.

## 4 Discussion

The decisive role of plant genotype for improving drought tolerance in *Lolium perenne* was reflected in higher RWC, dry weight and N content in S10 genotype under drought stress than those in Vigor and Speedy genotypes, indicating increased drought tolerance in this genotype of *Lolium*.

In this study, the accumulation of proline and some other amino acids increased in the three genotypes of *Lolium* under drought stress. However, the content of proline, aspartate, glutamate, phenylalanine, and tyrosine was higher in S10 genotypes than that in the other two genotypes (Fig 2). These results were consistent with the previous studies on tall fescue that showed up-regulation of proline and some amino acids under drought stress [36, 37]. Therefore, the results of this study indicate an increase in physiological response capacity of S10 genotype to drought stress, which may enable the plant to have excellent stomatal control and accordingly reduce water utilization.

Based on the *Brachypodium* protein database, we identified 467, 456, and 97 differentially abundant proteins in Vigor, Speedy, and S10 genotypes, respectively, under drought stress (S1 Table). These results demonstrate that drought stress coincides with changes in proteins. We also found that the number of differentially decreased proteins (57) was higher than that of differentially increased proteins (40) in the S10 genotype, showing that protein breakdown is the primary function during drought stress in this genotype. Some previous studies also showed that drought resistance was involved in an alteration in the gene expression responsive to a decrease in the transcript abundance [38, 39].

In the present study, we found that the increased proteins in the two commercial genotypes were mostly involved in carbon and energy metabolism, photosynthesis, TCA cycle, redox, and transport categories. However, in the S10 genotype, they were in N and amino acid metabolism category (Fig 5B, S1 Table). Also, in Vigor and Speedy genotypes, the decreased proteins were related to cell wall, N, and amino acid metabolism category and redox. However, in the S10 genotype, they were in carbon and energy metabolisms.

Preventing photosynthesis due to the closure of stomata is one of the destructive effects of drought stress which in turn results in a total decrease in the expression level of the proteins involved in photosynthesis [40, 41]. In contrast, in our study, the majority of photosynthesis-related proteins increased as an influence of drought stress in the two commercial genotypes. This increase in abundance mainly involved carbon fixation enzymes, including RuBisCO, RuBisCO activase, FBA, glyceraldehyde-3-phosphate dehydrogenase, sedoheptulose-1,7-bisphosphatase, and phosphoribulokinase. While light reaction-involved proteins were down-regulated under drought in all three genotypes of *Lolium*; therefore, the increase in carbon fixation enzymes may remain useless.

Damage to photosystems usually occurs when plants are subjected to drought stress [42]. This statement was confirmed in our study by the identification of reduction in light-harvesting complexes in Vigor and Speedy genotypes, and somehow in S10 genotype. The decrease in abundance of these polypeptides has been reported before in several species under drought stress [43–45].

All of the enzymes involved in the Krebs cycle were enhanced under drought stress in the two commercial genotypes. The up-regulation of the proteins involved in the Krebs cycle under drought stress is in accordance with the results of previous researches [46, 47]. Enhancing TCA cycle activity may ensure energy for various processes towards tolerance to drought stress. The TCA pathway is one of the main pathways of energy production, and the up-regulation of the enzymes involved in this pathway can be due to the cell's demand for energy.

Sucrose synthase (SS), fructose-1,6-bisphosphatase, and ADP-glucose pyrophosphorylase are the key enzymes involved in the regulation of sucrose synthesis in the cytoplasm and starch synthesis in the chloroplast. Our data showed that under drought stress, the abundance of SS in the S10 genotype increased to 3-fold compared to the control condition. However, the abundance of fructose-1,6-bisphosphatase was reduced in this genotype. SS belongs to a large family of glycosyltransferase enzymes in plants that catalyzes the synthesis of sucrose from fructose and UDP-glucose, providing carbon for respiration and the biosynthesis of starch and cellulose [48].

During drought stress, biosynthesis of starch is generally emptied in several plant species [49, 50]. The ADP-glucose pyrophosphorylase enzyme plays a crucial role in the biosynthesis regulation of starch from glucose-1-phosphate. In the genotype S10, we found a reduction in the abundance of the ADP-glucose pyrophosphorylase. In contrast, the abundance of this protein and starch synthase increased in the two commercial genotypes of *Lolium*. There is evidence that at the beginning of drought stress, a short-term increase in starch synthesis may happen [51]. Then, the up-regulated expression levels of starch-degradation enzymes could lead to a reduced level of starch in the plants.

On the one hand, the plant requires energy to cope with the stress; on the other hand, the rate of photosynthesis in the plant decreases under stress conditions. Therefore, to provide the required energy for various activated pathways against stress, the plant starts metabolizing starch. Also, cell wall invertase, which is involved in sucrose degradation, decreased under drought stress in the two commercial genotypes of *Lolium*. Invertase catalyzes the hydrolysis of sucrose into glucose and fructose, plays a crucial role in primary metabolism and plant development. Down-regulation of cell wall invertase and sucrose synthase was reported from *Piriformospora indica*-inoculated barley exposed to drought stress compared to non-inoculated plants [52]. Our results imply a crucial role of SS and invertase in *Lolium* genotypes in the regulation of sugar biosynthesis when exposed to drought stress. Since sucrose is involved in the stability of proteins as a compatible osmolyte, thus in stressed plants, enhancement of sucrose may play an essential regulatory effect in the alleviation of damage [53].

The amounts of most of the proteins in the two commercial genotypes were down-regulated at the cell wall level in order to cope with drought stress. Plants may down-regulate some enzymes, such as cell wall hydrolysates, in order to reduce the hydrolysis of cell wall polysaccharides, which may be an approach to save their energy and preserve their carbohydrate reserves, and thus to survive under stress. An increase in cell wall synthesis, under drought stress conditions, is probably due to the increased mechanical strength as a strategy to reduce dehydration [54].

Arabinogalactan proteins (AGPs) belonging to the hydroxyproline-rich cell wall glycoprotein superfamily, are implicated in various aspects of plant growth and development, including cell differentiation, cell expansion and abiotic stress response through modulating cell wall expansion [55] and possible interaction of the plant with microbial endophytes [56]. We identified FLA11 as a fasciclin-like arabinogalactan protein that was up-regulated in the S10 genotype under drought stress. Similarly, the up-regulation of five FLA genes in *Populus trichocarpa* was reported under salt stress [57]. Accumulation of AGPs in the S10 genotype could be stimulating for extreme amounts of cell walls expansion under drought stress. The enlargement of cell wall, in turn, can cause a considerably higher water absorption expanding the cell volume of plants.

In plants, phospholipases hydrolyze membrane phospholipids to phosphatidic acid (a membranous second messenger molecule); the target of phosphatidic acid is ABI1 that is implicated in ABA signaling in stomatal responses [58]. The latest studies demonstrate that phospholipase plays an essential role in plant drought stress tolerance [59, 60,61]. In this study, we found that phospholipases were considerably accumulated in the two commercial genotypes of *Lolium* under drought stress. The activity and transcript levels of PLD (phospholipase D) in cowpea and peanut increased under drought stress in cultivars that were drought susceptible compared to drought-tolerant cultivars [62, 63]. Hence, we propose that phospholipases may be implicated in the membrane rearrangement and first happenings in ABA signal transduction in stomatal movement in Vigor and Speedy genotypes under drought stress.

Various studies have reported that drought stress alters the volume of free amino acids in plants' cells [64, 36]. These alterations can be due to increased protein degradation under stress [65, 66]. The participation of metabolic pathways of amino acids in growth and regulation of tolerance and adaption to stresses has been shown in plants. In this study, drought stress enhanced synthesis and degradative pathways of some amino acids, especially methionine, cysteine, aspartate, glycine, and proline. It strongly enhanced the synthesis of proline, as biochemical data also confirm that proline content was up-regulated in drought-stressed plants in all three genotypes (Table 1). Drought stress can induce proteolysis of protein and thereby leads to an accumulation of amino acids [67]. In our study, drought led to a rise in amino acid content in all three genotypes. The difference in amino acid contents resulted from drought stress is more likely to reflect the turnover of amino acids. The accumulation of P5CR, which participates in proline biosynthesis, was significantly higher under drought stress in the two commercial genotypes. Notwithstanding with no overall change at the protein level of P5CS in the examined genotypes, our biochemical analysis showed enhancement of proline level in leaves of all three genotypes. In the S10 genotype, we found a more significant increase than 2.1-fold in the accumulation of this amino acid, but only 1.2-fold alteration was recorded for Vigor and Speedy genotypes (Table 1).

Drought stress decreased photosynthesis, coupled with the reduction in nitrogen metabolism [68, 69]. Nitrogen assimilation occurs in plants via two key enzymes, glutamine synthetase (GS) and glutamate synthase (GOGAT). Singh et al. [70] showed that soil drying decreased the activities of these enzymes together with a decrease in N absorption and content of nitrogenous compounds in leaves of sunflower seedlings. Our results show that the abundance of GS and GOGAT decreased in the two commercial genotypes under stress, but no change was observed in the abundance of these two enzymes in the S10 genotype. Similarly, Ghaffari et al. [52] reported that drought stress declined NR, GS, and GOGAT enzymes in non-inoculated barley, but this was not the case for inoculated plants with *Piriformospora indica*. It was demonstrated in numerous studies that leaf nitrogen reduces progressively as the drought proceeds, and this can be correlated to photosynthetic machinery damage [71, 69]. Our biochemical data show that total nitrogen content decreased in Vigor and Speedy genotypes under drought stress, which confirms the lower activity of GS and GOGAT enzymes in this condition. Meanwhile, total nitrogen content did not change in the S10 genotype during drought stress, which is in accordance with the proteomic data. Numerous studies have reported crop productivity reduction due to drought stress as one of the most significant challenges [72, 73]. One of the reasons for the decreased growth rate of the plants exposed to drought stress can be due to the prevention of nitrogen fixation by drought stress. In this study, a down-regulation in the expression level of nitrite reductase (NR) protein was also detected in the two genotypes of Speedy and Vigor. The relative reduction of shoot dry weight under drought stress was similar for these two genotypes (about 11%), probably showing a lower NR activity in both genotypes. However, this reduction for the S10 genotype under drought was about 5.4 times smaller than the reduction in the two commercial genotypes.

Abscisic acid (ABA) is a phytohormone that plays a crucial role in plant tolerance to drought stress. ABA regulates many aspects of plant development under abiotic stresses, permitting the plant to resist drought conditions. Drought-induced increase in ABA content usually results in leaf stomata closure, stimulation of stress-related proteins, and provides different metabolites for the response to stress [74]. An increased ABA level has been formerly informed in several species under drought stress [20, 75,36]. We found an approximately 6-fold increase of ABA concentration in the two genotypes of Speedy and Vigor two days after drought stress. The ABA content is controlled by the relative values of biosynthesis, catabolism, conjugation, and its distribution through the plant. In our results, both the content of endogenous ABA and the expression of rate-limiting ABA biosynthetic protein (NCED) increased in the two commercial genotypes under drought stress. Similar proteomic analysis findings have been documented for several plant species, for instance, in *Phaseolus* [76], *Arabidopsis* [77], and *Stipa* [20] showing the up-regulation of *NCED* genes under drought stress. However, our findings showed that although no protein abundance changes of ABA biosynthesis-related enzymes were identified in the S10 genotype, the endogenous ABA increased in the leaves of this genotype, suggesting that the increase could be possibly due to the decreased catabolism of this hormone.

ROSs are generated as a routine by-product of aerobic metabolism in plant cells. Besides, drought stress is recognized to accelerate the accumulation of ROS in plant cells, lastly resulting in cell damage. Therefore, ROS levels need to be actively controlled for the protection of plants from drought-induced oxidative stress. The redox signaling also regulates a large number of transcripts that are necessary for stress acclimatization. Ascorbic acid (AsA), which is a constituent of the AsA-glutathione (GSH) cycle [78], maintains redox homeostasis in the plant cell. In order to maintain the antioxidative potential of AsA, rapid restitution of AsA is arranged by DHAR and MDHAR [78–80]. Activated enhancement of DHAR and MDHAR in these stress responses has been found in various plants. In our study, up-regulation of DHAR and MDHAR in the two commercial genotypes of *Lolium* under stress suggests that these genotypes may increase AsA content by increasing DHAR and MDHAR enzymes. Also, glutathione (GSH; γ-glutamyl-cysteinyl-glycine) plays an essential function in sequestration and transport of reduced sulfur participating directly or indirectly in the detoxification of ROS [81].

Regarding glutathione-related proteins, we identified that glutathione reductase and glutathione peroxidase were up-regulated only in commercial genotypes; but not in the S10 genotype. However, the up-regulation of glutathione S-transferase was observed in all three genotypes. We observed that reduced glutathione content increased in the three genotypes of *Lolium*, probably due to the action of glutathione-related proteins. Thioredoxins (Trxs) are well-conserved disulfide reductases that regulate the redox state of target proteins. In plants, Trxs are proved to be essential for plant tolerance to oxidative stresses [82]. In Vigor and Speedy genotypes, the up-regulation of thioredoxin M-type was detected under drought stress. Peroxiredoxins (Prx) are a family of thiol dependent peroxidases found in plant cells, which use thioredoxin as their electron donor for the catalysis of H_2_O_2_. They can act as redox sensors, signal transducers, and molecular chaperones through the alterations in their oligomeric structures [83]. Also, we found up-regulation of the two isoforms of peroxiredoxin type 2 in Vigor and Speedy genotypes under drought stress. Generally, high numbers of detected leaf proteins related to ROS scavengings such as APX, SOD, GR and CAT, glutathione peroxidase, and peroxiredoxin and thioredoxin reductase were up-regulated. Up-regulation of most antioxidant enzymes has been formerly documented in different species under drought stress [20, 84,47]. However, in the S10 genotype under drought stress, the antioxidant system did not respond as vigorously as expected, except for SOD and GST enzymes, which were enhanced to play defensive roles against ROS. Ghaffari et al. [52] reported that four isoforms of GST and three isoforms of ascorbate peroxidase were up-regulated only in the non-inoculated barley; but not in the inoculated ones with mycorrhiza. Glutathione S-transferase was also up-regulated in all three genotypes of *Lolium*. Possibly because of the up-regulation of ROS-generating proteins in commercial genotypes, it seems that the amount of produced ROS in these genotypes was higher than that in the S10 genotype. Thus they increased the number of activated enzymes in order to maintain their homeostasis.

The up-regulated proteins included heat shock proteins (HSPs) and aquaporins (AQP), which are known to be critical for stress acclimatization [85] and water transport [86]. Heat shock proteins are implicated in plant acclimatization to different stresses [85, 87]. Members of various heat shock protein families play substantial roles in trafficking and folding of proteins and assembling of macromolecular in the cell [88]. Our results showed induction of heat shock protein 60, and increased abundance was found for the chaperonin-60ALPHA, chaperonin 20, and DNAJ, which is named HSP40, under drought treatment only in the two commercial genotypes. The expression of HSP has been indicated to increase in many plant species under drought stress [89, 20, 84]. Conversely, Ghaffari et al. [52] have reported that under drought stress, the expression of HSPs decreased in the non-mycorrhizal plants of barley. Our findings highlight that the HSPs may play an essential role in maintaining protein stability in some genotypes under drought stress.

Strikingly, in the two genotypes of Vigor and Speedy under drought stress, five plasma membrane intrinsic proteins were found, and all these exhibited increased levels. However, these proteins in the S10 genotype remained unchanged under stress. Several investigators have formerly found the up-regulation of aquaporins in response to water stress in different plant species [90–92]. Also, the transcript level of aquaporin was enhanced by *Trichoderma harzianum* colonization in rice [93].

In our study, the evaluation of RWC content during drought stress indicated significantly higher values of RWC in the S10 genotype in comparison to the two commercial genotypes (Table 1). The better water status of the S10 genotype ensured that the expression of aquaporins gene is unnecessary in this genotype and permitted to maintain higher leaf net photosynthesis and water use efficiency than the two commercial genotypes when exposed to drought stress.

Comparably, low genetic gains characterize current population-based breeding methods for essential traits such as biomass yield in many plants. However, grass species have adopted a range of various breeding systems, some promoting self-pollination, some cross-pollination, and some asexual propagation. Our results demonstrated that the self-pollinating genotype of *Lolium perenne* (S10) might provide a better buffer in response to drought stress. Therefore, self-pollinating genotypes of the plant might be advantageous over cross-pollinating ones due to having more gain for desired traits, including drought stress resistance after fixation in genetic loci and selection for the well-performed characteristics [13]. In our study, it seems that the S10 genotype is a potential new genetic resource with a self-pollinating reproductive system that could be introduced to different genetic backgrounds for better resistance to biotic and abiotic stresses.

## 5 Conclusion

**Fig 7:**
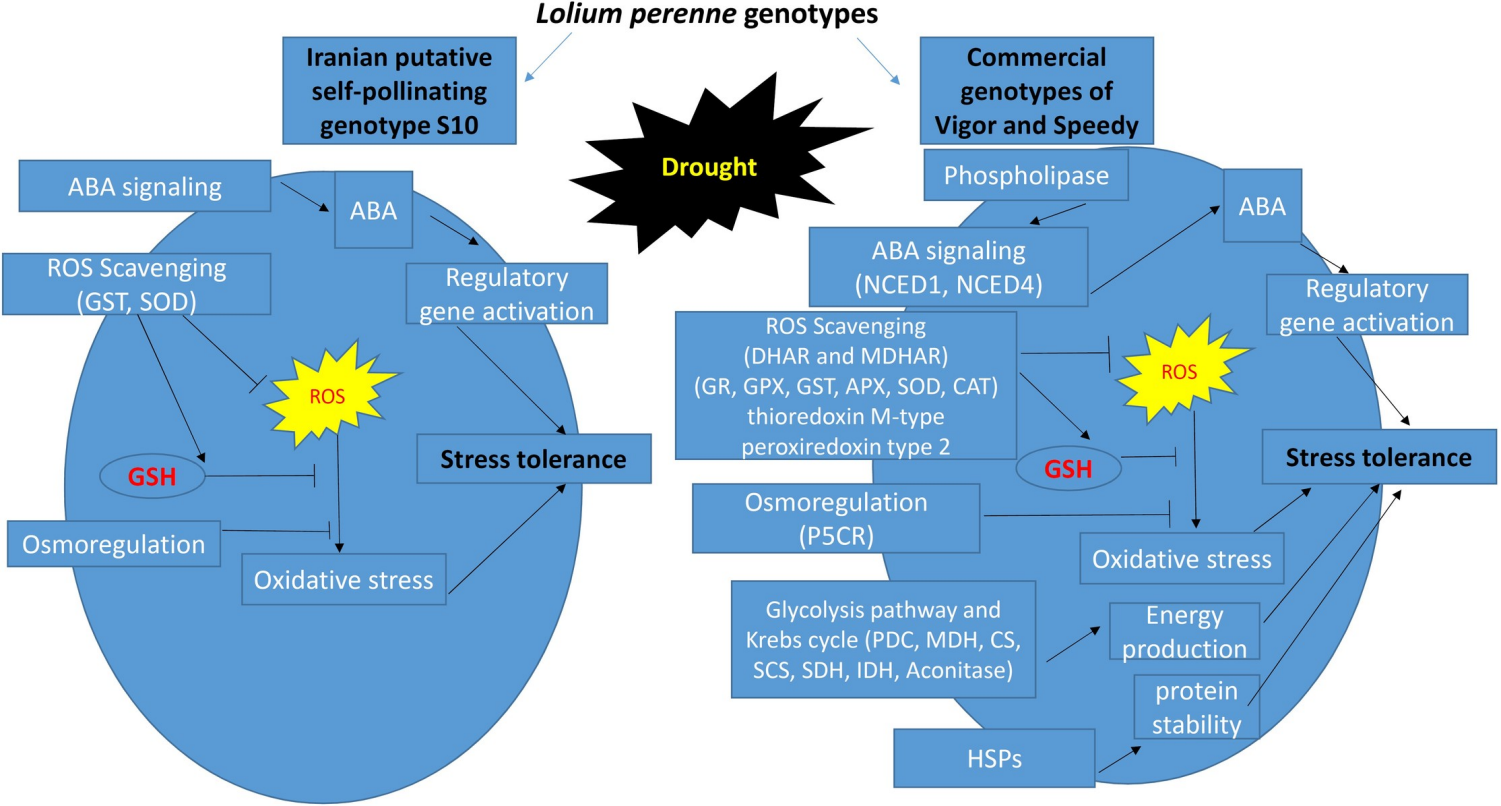
A schematic diagram of differences in drought response strategies between the S10 genotype and Vigor and Speedy genotypes of perennial ryegrass. Abbreviations: **PDC**: pyruvate dehydrogenase complex, **MDH**: malate dehydrogenase, **CS**: citrate synthase, **SCS**: succinyl-CoA ligase, **SDH**: succinate dehydrogenase and **IDH**: isocitrate dehydrogenase.

This study provides perceptions into the effects of drought stress on the abundance of proteins in the leaves of three genotypes of *Lolium perenne*. The results of this study indeed showed that RWC and SDW, proline, ABA, N, and amino acid contents, and antioxidant enzyme activities were significantly higher in the drought-tolerant genotype of S10 under drought stress in comparison with the two commercial genotypes. Results of the proteomics analysis revealed significant differences between the examined genotypes of *Lolium* under drought stress conditions (Fig 7), and this result seems to be related to the higher ability of the S10 genotype in response to drought stress. Intriguingly, we found only slight alterations in the protein profiles of S10 genotypes under drought stress, proposing that protein abundance was less influenced, possibly due to the tolerance in this genotype. That consequence may be related to the self-compatibility mechanism that can improve grass characteristics and alleviate some adverse effects of drought stress. Also, the results showed that acclimatization to drought stress in the two commercial genotypes involved specific responses that resembled between Vigor and Speedy. In this study, some underlying mechanisms were identified through which the plant responses may be improved to drought. These mechanisms included (1) significant increases in the abundance of antioxidant enzymes to scavenge ROS; and (2) changes in the expression of various proteins, including those involved in TCA cycle, amino acid and ABA metabolisms, aquaporins, and HSPs. These changes increased stress tolerance through ABA-mediated regulation of stomatal control to diminish water loss and assist in decreasing the adverse effects of drought by inhibiting damage to fundamental cell building blocks and improving osmoregulation. Additional analyses may be also required to clarify the root proteome that seems to participate in this response.

## Supporting information

S1 Table Proteins showing significant changes in responses to drought stress in Vigor, Speedy, and S10 genotypes. B.C. is the abbreviation of the Bin Code (i.e., major functional categories). No.: identification number. ID: Δ: fold changes in drought-stressed plants (D) with respect to the control ones (C) (up: D/C, down:—C/D). new: not present in C; D.: disappeared, not present in drought stress.
(DOCX)

S1 Fig One-dimensional SDS-Polyacrylamide Gel Electrophoresis (1D SDS-PAGE) of total protein of three genotypes of *Lolium perenne*. Left to right: Protein marker (lane 1), Control sample of Vigor genotype (lane 2,3), Drought sample of Vigor genotype (lane 4,5), Drought sample of Speedy genotype (lane 6), Control sample of Speedy genotype (lane 7), Control sample of S10 genotype (lane 8,9), Drought sample of S10 genotype (lane 10, 11).
(TIF)

S1 Data (XLSX)
