# Supplementary material for: Comparative physiological and proteomic analysis indicates lower shock response to drought stress conditions in a self-pollinating perennial ryegrass
Source: PLoS One. 2020 Jun 18;15(6):e0234317. doi: 10.1371/journal.pone.0234317 (PMC7302502; doi:10.1371/journal.pone.0234317)
Supplement: S1 Table — B.C. is the abbreviation of the Bin Code (i.e., major functional categories). No.: identification number. ID: Δ: fold changes in drought-stressed plants (D) with respect to the control ones (C) (up: D/C, down:—C/D). new: not present in C; D.: disappeared, not present in drought stress. (DOCX) [file pone.0234317.s001.docx]

| **B.C.** | **No.** | **Name** | **ID** | **Δ:D/C, genotype** | | |
| --- | --- | --- | --- | --- | --- | --- |
|  |  |  |  | **Vigor** | **Speedy** | **S10** |
| **Carbon and energy metabolism (1, 2, 3, 4, 5,6,7,8,9, 25)** | | | | | | |
| **1** | 38 | Chlorophyll a-b binding protein, chloroplastic | bradi3g07190.1 | 1.676241 | - | - |
| **1** | 455 | photosystem II reaction center PsbP family protein | bradi4g04640.1 | -2.56963 | - | - |
| **1** | 560 | PPL2 (PsbP-like protein 2) | bradi1g66296.1 | 7.669556 | - | - |
| **1** | 19 | cytochrome f apoprotein | bradi3g27332.1 | 1.519148 | - | - |
| **1** | 5 | ATPase alpha subunit | bradi5g08886.1 | 2.049873 | - | - |
| **1** | 169 | NAD(P)H-quinone oxidoreductase subunit H, chloroplastic | bradi3g27266.1 | 3.147124 |  | - |
| **1** | 53 | PsaC subunit of photosystem I | bradi1g05756.1 | -894.135 | - | - |
| **1** | 85 | PSAE-2 (photosystem I subunit E-2) | bradi1g29000.1 | -2.63924 | - |  |
| **1** | 215 | oxidoreductase NAD-binding domain-containing protein | bradi2g24210.2 | 1.798674 | - | - |
| **1** | 48 | glycerate dehydrogenase | bradi3g00330.1 | 1.942647 | - | - |
| **1** | 244 | phosphoribulokinase | bradi2g46690.3 | 2.077212 | - | - |
| **1** | 118 | triose-phosphate isomerase | bradi4g36310.1 | 1.578445 | - | - |
| **1** | 15 | Fructose-bisphosphate aldolase (chl) | bradi4g24367.1 | 1.624623 | - | - |
| **1** | 3 | RCA (RUBISCO Activase) | bradi4g09125.2 | 1.753148 |  | - |
| **1** | 26 | Glyceraldehyde-3-phosphate dehydrogenase | bradi5g12330.1 | 1.704608 | - | - |
| **3** | 609 | aldo/keto reductase family protein | bradi1g58220.1 | 3.048479 | - | - |
| **3** | 823 | Alpha-galactosidase | bradi1g17730.1 | -10.4742 |  | - |
| **7** | 423 | Glucose-6-phosphate 1-dehydrogenase | bradi1g60050.1 | 2.11659 | - | - |
| **7** | 667 | transaldolase | bradi2g59370.1 | -3.71989 | - | - |
| **8** | 561 | fumarate hydratase | bradi1g63020.1 | -2.34936 | - | - |
| **25** | 526 | 4-hydroxy-4-methyl-2-oxoglutarate aldolase | bradi1g29550.1 | -2.13366 | - | - |
| **1** | 4 | PS.lightreaction.photosystem II.LHC-II | bradi1g12130.1 | - | -2.08832 | - |
| **1** | 32 | PS.lightreaction.photosystem II.LHC-II | bradi1g24760.1 | - | -3.26915 | - |
| **1** | 67 | PS.lightreaction.photosystem II.LHC-II | bradi1g23610.2 | - | -3.84442 | - |
| **1** | 207 | PS.lightreaction.photosystem II.LHC-II | bradi4g37210.1 | - | D | - |
| **1** | 144 | PS.lightreaction.photosystem II.PSII polypeptide subunits | bradi3g17640.2 | - | 1.574311 | - |
| **1** | 235 | PS.lightreaction.photosystem II.PSII polypeptide subunits | bradi1g77047.1 | - | -2.50196 | - |
| **1** | 47 | PS.lightreaction.photosystem I.PSI polypeptide subunits | bradi1g06890.1 | - | -4.4515 | - |
| **1** | 88 | PS.lightreaction.photosystem I.PSI polypeptide subunits | bradi1g06890.2 | - | -5.18164 | - |
| **1** | 506 | PS.lightreaction.photosystem II.PSII polypeptide subunits | bradi4g40710.1 | - | -3.54445 | - |
| **1** | 164 | PS.lightreaction.photosystem I.PSI polypeptide subunits | bradi1g06890.3 | - | -2.19566 | - |
| **1** | 803 | PS.lightreaction.cytochrome b6/f | bradi1g06890.4 | - | D | - |
| **1** | 72 | PS.lightreaction.cytochrome b6/f.iron sulfur subunit | bradi1g06890.5 | - | -2.16394 | - |
| **1** | 382 | PS.lightreaction.cyclic electron flow-chlororespiration | bradi1g52550.7 | - | -2.62169 | - |
| **1** | 424 | PS.lightreaction.NADH DH | bradi5g17027.1 | - | -2.62894 | - |
| **1** | 112 | PS.calvin cycle.phosphoglycerate kinase | bradi3g05220.1 | - | 1.606817 | - |
| **1** | 912 | PS.calvin cycle.transketolase | bradi5g07190.1 | - | new | - |
| **1** | 24 | sedoheptulose-bisphosphatase | bradi2g55150.1 | - | 2.56503 | - |
| **1** | 36 | PS.calvin cycle.rubisco small subunit | bradi3g26391.2 | - | 2.601254 | - |
| **2** | 788 | Starch synthase 1 | bradi1g48610.1 | - | 2.478235 | - |
| **2** | 286 | major CHO metabolism.degradation.sucrose., Fructokinase I | bradi2g57500.1 | - | 1.953845 | - |
| **3** | 180 | minor CHO metabolism.others | bradi1g58210.1 | - | 2.057853 | - |
| **3** | 719 | minor CHO metabolism.others | bradi3g37327.1 | - | new | - |
| **4** | 106 | glycolysis.cytosolic branch.UGPase | bradi4g37350.1 | - | 1.617126 | - |
| **2** | 390 | major CHO metabolism.degradation.sucrose.Hexokinase I | bradi2g19400.3 | - | 1.635821 | - |
| **4** | 594 | glycolysis.cytosolic branch.pyruvate kinase (PK) | bradi5g26140.1 | - | 1.98627 | - |
| **4** | 422 | glycolysis.plastid branch.glucose-6-phosphate isomerase | bradi4g32530.1 | - | 2.829449 | - |
| **6** | 648 | gluconeogenesis / glyoxylate cycle.Malate DH | bradi3g37140.1 | - | 7.344565 | - |
| **6** | 721 | gluconeogenesis / glyoxylate cycle.isocitrate lyase | bradi5g07880.1 | - | 9.105815 | - |
| **7** | 642 | OPP.oxidative PP.6-phosphogluconate dehydrogenase | bradi3g46080.1 | - | 1.775471 | - |
| **7** | 236 | OPP.non-reductive PP.ribose 5-phosphate isomerase | bradi1g55740.2 | - | -3.80213 | - |
| **8** | 666 | TCA / org transformation.TCA.pyruvate DH.E2 | bradi3g36520.1 | - | -2.94335 | - |
| **8** | 161 | TCA / org transformation.TCA.aconitase | bradi3g15050.1 | - | 2.159319 | - |
| **8** | 368 | TCA/org transformation.TCA.succinyl-CoA ligase | bradi3g49070.1 | - | 1.968666 | - |
| **8** | 60 | TCA/org transformation.carbonic anhydrases | bradi2g44856.1 | - | -3.57747 | - |
| **9** | 408 | mitochondrial electron transport / ATP synthesis.NADH-DH.complex I | bradi1g53070.1 | - | 2.892643 | - |
| **9** | 321 | mitochondrial electron transport / ATP synthesis.NADH-DH.localisation not clear | bradi1g11520.2 | - | 2.413694 | - |
| **9** | 433 | mitochondrial electron transport / ATP synthesis.NADH-DH.localisation not clear | bradi3g55340.1 | - | 1.677801 | - |
| **9** | 371 | mitochondrial electron transport / ATP synthesis.cytochrome c reductase | bradi1g54370.1 | - | -3.08925 | - |
| **9** | 469 | mitochondrial electron transport / ATP synthesis.cytochrome c reductase | bradi3g44410.1 | - | 2.023088 | - |
| **9** | 23 | mitochondrial electron transport / ATP synthesis.F1-ATPase | bradi2g46790.2 |  | 1.559004 | - |
| **9** | 57 | mitochondrial electron transport / ATP synthesis.F1-ATPase | bradi2g13090.1 |  | 1.602017 | - |
| **9** | 156 | mitochondrial electron transport / ATP synthesis.F1-ATPase | bradi3g24730.1 |  | 3.262183 | - |
| **9** | 394 | mitochondrial electron transport / ATP synthesis.F1-ATPase | bradi3g02890.1 |  | 2.451356 | - |
| **9** | 482 | mitochondrial electron transport / ATP synthesis.F1-ATPase | bradi3g53000.1 |  | 1.846225 | - |
| **25** | 556 | C1-metabolism | bradi4g31680.1 | - | 23.93588 | - |
| **1** | 25 | PS.lightreaction.photosystem II.LHC-II | bradi4g07380.1 | - | - | 3.175224 |
| **1** | 457 | PS.lightreaction.photosystem I.LHC-I | bradi2g42300.1 | - | - | -2.477 |
| **1** | 99 | PS.lightreaction.ATP synthase.subunit B (ATPF) | bradi5g08883.1 | - | - | -2.74173 |
| **2** | 489 | SUS4; UDP-glycosyltransferase/ sucrose synthase | bradi1g46670.3 | - | - | 2.950689 |
| **8** | 753 | TCA / org transformation.other organic acid transformatons.atp-citrate lyase | bradi4g04390.1 | - | - | 3.39989 |
| **9** | 791 | mitochondrial electron transport / ATP synthesis.cytochrome c oxidase | bradi1g60710.2 | - | - | 2.446803 |
| **1** | 294 | APE1 (ACCLIMATION OF PHOTOSYNTHESIS TO ENVIRONMENT) | bradi3g22030.1 | 3.540277 | 1.561751 | - |
| **1** | 90 | Chlorophyll a-b binding protein, chloroplastic | bradi3g42620.1 | -6.51518 | -3.24525 | - |
| **1** | 6 | CP47, subunit of the II reaction center | bradi1g05740.1 | 2.651058 | 1.630391 | - |
| **1** | 9 | Photosystem II CP43 reaction center protein | bradi1g78082.1 | 2.972099 | 3.078445 | - |
| **1** | 10 | Photosystem II D2 protein | bradi1g78076.1 | 2.760612 | 2.255155 | - |
| **1** | 17 | PSBA \| Encodes chlorophyll binding protein D1, a part of the photosystem II reaction center core \| | bradi5g10966.1 | 2.415535 | 1.705336 | - |
| **1** | 228 | PS.lightreaction.photosystem II.PSII polypeptide subunits | bradi3g39830.1 | -3.91516 | -28.4982 | - |
| **1** | 31 | PSAA \| Encodes psaA protein comprising the reaction center for photosystem I along with psaB protein | bradi5g23845.1 | 5.947117 | 3.685968 | - |
| **1** | 847 | PSAH2, PSAH-2, PSI-H \| PSAH2 (Photosystem I SUBUNIT H2) | bradi2g16810.1 | D | D | - |
| **1** | 636 | STN7 \| STN7 (Stt7 homolog STN7); kinase/ protein kinase | bradi2g17660.1 | 2.275846 | 1.672034 | - |
| **1** | 16 | ATPA \| Encodes the ATPase alpha subunit | bradi5g23842.1 | 1.529961 | 1.534743 | - |
| **1** | 226 | PGR5-LIKE A \| PGR5-LIKE A | bradi1g00940.1 | 3.343806 | 1.638358 | - |
| **1** | 465 | NDH-O \| NDH-O (NAD(P)H:plastoquinone dehydrogenase complex subunit O) | bradi2g61410.1 | 1.644896 | -4.72291 | - |
| **1** | 69 | Serine hydroxymethyltransferase | bradi1g09300.2 | 3.371445 | 1.829602 | - |
| **1** | 1 | RBCL, large subunit of RUBISCO | bradi3g27325.1 | 1.60334 | 1.634211 | - |
| **1** | 22 | Ribulose bisphosphate carboxylase small chain | bradi5g04080.1 | 1.926567 | 2.13781 | - |
| **1** | 34 | glyceraldehyde-3-phosphate dehydrogenase (Chl) | bradi1g76470.1 | 2.149099 | 2.319031 | - |
| **1** | 503 | Fructose-bisphosphate aldolase (Chl) | bradi2g01350.1 | -3.0881 | -5.43471 | - |
| **2** | 896 | Starch-related R1 protein | bradi1g41907.2 | new | new | - |
| **3** | 64 | mannose 6-phosphate reductase (NADPH-dependent) | bradi3g02340.1 | 3.942408 | 5.071367 | - |
| **3** | 289 | aldo/keto reductase family protein | bradi1g14710.1 | 1.75192 | 1.646331 | - |
| **3** | 911 | aldose 1-epimerase | bradi4g28160.1 | new | new | - |
| **3** | 716 | Alpha-galactosidase | bradi3g29810.3 | -6.50894 | -3.24428 | - |
| **4** | 300 | Glucose-6-phosphate isomerase | bradi2g37400.2 | 1.743434 | 1.543041 | - |
| **1** | 40 | Transketolase | bradi1g51670.1 | 1.777847 | 1.681398 | - |
| **2** | 632 | ADP-glucose pyrophosphorylase | bradi4g27570.2 | new | 1.939504 | - |
| **2** | 645 | cell wall invertase | bradi3g44990.2 | -5.01478 | -7.4971 | - |
| **2** | 708 | sucrose cleavage protein-like | bradi3g56460.1 | -2.33037 | D | - |
| **5** | 282 | 3-chloroallyl aldehyde dehydrogenase | bradi3g36150.2 | 3.895472 | 1.987293 | - |
| **6** | 102 | Malate dehydrogenase | bradi1g07170.2 | 2.039636 | 1.674917 | - |
| **6** | 491 | gluconeogenesis / glyoxylate cycle.pyruvate dikinase | bradi2g25745.1 | 3.681232 | 2.07753 |  |
| **7** | 375 | Oxidoreductase | bradi2g07650.1 | 1.846681 | -2.55844 | - |
| **8** | 397 | Pyruvate dehydrogenase E1 component subunit beta | bradi3g41480.3 | 1.537349 | 3.222228 | - |
| **8** | 603 | Pyruvate dehydrogenase E1 component subunit beta | bradi4g34810.1 | 2.829007 | 4.474047 | - |
| **8** | 309 | Citrate synthase | bradi3g06930.1 | 1.888595 | 2.527388 | - |
| **8** | 336 | Aconitate hydratase | bradi1g75960.2 | 2.185134 | 2.651682 | - |
| **8** | 539 | isocitrate dehydrogenase | bradi5g13490.1 | new | 2.360774 | - |
| **8** | 565 | succinyl-CoA ligase | bradi1g23600.1 | 5.315558 | 18.87783 | - |
| **8** | 412 | Succinate dehydrogenase [ubiquinone] flavoprotein subunit, mitochondrial | bradi1g07020.1 | 2.758752 | 2.369547 | - |
| **8** | 682 | Malic enzyme | bradi1g27000.1 | 3.018073 | new | - |
| **8** | 553 | Isocitrate dehydrogenase [NAD] subunit, mitochondrial | bradi3g43300.1 | 1.6946 | 1.955641 | - |
| **9** | 878 | mitochondrial ATP synthase g subunit | bradi2g42680.1 | D | new | - |
| **25** | 537 | Formate dehydrogenase, mitochondrial | bradi1g42270.1 | 1.503111 | 3.86486 | - |
| **25** | 777 | Methylenetetrahydrofolate reductase | bradi1g04140.1 | -2.10878 | 2.092876 | - |
| **9** | 786 | NADH-ubiquinone oxidoreductase | bradi3g34360.1 | -3.68625 | -2.56905 | - |
| **1** | 54 | D1 subunit of photosystem I and II reaction centers | bradi5g23844.1 | 7.084402 | - | -4.23532 |
| **1** | 843 | PSAK (photosystem I subunit K) | bradi1g58350.1 | -1.59419 | - | D |
| **1** | 142 | ATPase epsilon subunit | bradi5g05674.1 | 1.923539 | - | -3.96558 |
| **1** | 33 | ATPC1 | bradi1g26350.1 | 2.122859 | - | -2.89078 |
| **1** | 42 | glycolate oxidase | bradi1g06430.1 | 1.911106 | - | -2.32205 |
| **1** | 71 | glycolate oxidase | bradi1g58480.1 | 2.33393 | - | -2.07373 |
| **1** | 11 | Phosphoglycerate kinase | bradi2g21120.1 | 1.939728 |  | -2.54878 |
| **1** | 83 | fructose-1,6-bisphosphatase | bradi1g67020.1 | 1.741578 | - | -2.1909 |
| **1** | 127 | fructose-1,6-bisphosphatase | bradi2g56030.1 | 2.851399 | - | -2.08776 |
| **2** | 198 | ADP-glucose pyrophosphorylase | bradi1g09537.1 | 2.139848 | - | -2.34739 |
| **9** | 2 | ATP synthase subunit beta | bradi2g20977.1 | 1.638661 | - | -2.44845 |
| **1** | 213 | PS.lightreaction.photosystem II.LHC-II | bradi4g30060.2 | - | -4.68508 | -2.38262 |
| **1** | 63 | PS.lightreaction.photosystem II.PSII polypeptide subunits | bradi5g23866.1 | - | -2.5263 | -3.55498 |
| **1** | 901 | PS.lightreaction.cyclic electron flow-chlororespiration | bradi1g74570.1 | - | 2.80505 | new |
| **8** | 617 | TCA / org transformation.other organic acid transformatons.misc | bradi4g31310.1 | - | 9.193307 | 8.882547 |
| **1** | 186 | Fructose-bisphosphate aldolase (cyto) | bradi1g36260.1 | 2.003792 | 1.804895 | -3.04297 |
| **2** | 176 | ADP-glucose pyrophosphorylase | bradi3g22330.1 | 1.580713 | 2.904941 | -2.33373 |
| **2** | 676 | Alpha-1,4 glucan phosphorylase (starch phosphorylase) | bradi2g55120.1 | 1.829129 | 1.558683 | 1.530686 |
| **5** | 693 | 3-chloroallyl aldehyde dehydrogenase | bradi5g12617.1 | 3.84442 | 2.64945 | 2.413262 |
| **8** | 569 | Pyruvate dehydrogenase E1 component subunit alpha | bradi3g59750.1 | 1.55372 | 3.14639 | - |
| **8** | 887 | NADP-malic enzyme 1 | bradi2g49540.1 | new | New | new |
| **Cell wall (10)** |  |  |  |  |  |  |
| **10** | 885 | NAD-dependent epimerase | bradi1g66440.1 | new | - | - |
| **10** | 872 | Cellulase | bradi5g16557.1 | D | - | - |
| **10** | 584 | Pectinesterase | bradi2g11860.1 | -8.29411 | - | - |
| **10** | 689 | UDP-glucose 6-dehydrogenase | bradi1g08120.1 | -3.57569 | 2.096863 | - |
| **10** | 327 | beta -1,4-glucanase | bradi1g08560.1 | -3.80106 | -2.83146 | - |
| **10** | 439 | xylan 1,4-beta-xylosidase | bradi5g23470.1 | -8.29677 | -2.63727 | - |
| **10** | 835 | polygalacturonase (pectinase) | bradi4g11087.1 | -8.1264 | -6.20741 | - |
| **10** | 875 | GDP-mannose 3,5-epimerase | bradi3g26860.4 | 1.887139 | - | - |
| **10** | 857 | arabinogalactan-rich proteins (AGPs)  (FLA11) | bradi2g00220.1 | D | D | 5.151994 |
| **10** | 208 | Cellulase | bradi1g08570.1 | -5.59551 | -2.80628 | - |
| **Lipid metabolism (11)** |  |  |  |  |  |  |
| **11** | 314 | enoyl-[acyl-carrier-protein] reductase (NADH) | bradi1g37040.1 | -4.24348 | - | - |
| **11** | 524 | sulfotransferase | bradi3g34340.1 | 1.581529 | - | - |
| **11** | 678 | quinone reductase family protein | bradi1g59450.1 | -2.69037 | - | - |
| **11** | 818 | triacylglycerol lipase | bradi5g15300.1 | 3.117979 | - | - |
| **11** | 687 | glycerophosphodiester phosphodiesterase/ kinase | bradi3g47340.1 | -4.02399 | - | - |
| **11** | 745 | lipid metabolism.FA synthesis and FA elongation.ACP protein | bradi1g62310.1 | - | D | - |
| **11** | 817 | lipid metabolism.FA synthesis and FA elongation.ACP desaturase | bradi2g58930.1 | - | D | - |
| **11** | 724 | lipid metabolism.lipid degradation.beta-oxidation.multifunctional | bradi3g10180.3 | - | new | - |
| **11** | 836 | lipid metabolism.FA synthesis and FA elongation.Acetyl CoA Carboxylation | bradi3g24417.1 | - | - | 1.53956 |
| **11** | 795 | protein malonyl CoA-acyl carrier protein transacylase | bradi1g65140.2 | new | D | - |
| **11** | 555 | FQR1 (FLAVODOXIN-LIKE QUINONE REDUCTASE 1);FMN binding/oxidoreductase | bradi2g20710.1 | -4.53286 | -3.31743 | - |
| **11** | 600 | Phospholipase D | bradi2g34290.1 | 2.158365 | 3.221503 | - |
| **11** | 903 | Phospholipase | bradi2g04650.1 | new | 3.522037 | - |
| **11** | 656 | phosphodiesterase | bradi3g39790.1 | -2.94768 | -2.72991 | - |
| **N and amino acid metabolism (12, 13)** | | | | | | |
| **12** | 681 | Glutamate dehydrogenase | bradi1g05680.1 | -4.24938 | - | - |
| **13** | 174 | Glutamate decarboxylase | bradi3g37830.1 | 1.76949 | - | - |
| **13** | 94 | ATMS1; 5-methyltetrahydropteroyltriglutamate-homocysteine S-methyltransferase/ methionine synthase | bradi1g13290.1 | -2.05929 | - | - |
| **13** | 383 | Cysteine synthase | bradi5g00610.1 | 2.789887 | - | - |
| **13** | 466 | L,L-diaminopimelate aminotransferase | bradi1g47650.1 | 1.644255 | - | - |
| **13** | 644 | glyoxalase I family protein | bradi1g13190.1 | -3.3505 | - | - |
| **13** | 826 | acetyl-CoA C-acyltransferase | bradi3g55420.1 | 1.500792 | - | - |
| **12** | 114 | Glutamate synthase (GOGAT) | bradi1g19080.1 | -2.4765 | -3.87706 | - |
| **13** | 547 | amino acid metabolism.synthesis.aspartate family aspartate semialdehyde dehydrogenase | bradi1g14590.1 | - | 2.959192 | - |
| **13** | 157 | amino acid metabolism.synthesis.aspartate family.methionine | bradi4g01200.2 | - | 1.521601 | - |
| **13** | 363 | ketol-acid reductoisomerase | bradi2g45330.1 | - | 2.163983 | - |
| **13** | 720 | amino acid metabolism.synthesis.serine-glycine-cysteine group.serine.phosphoglycerate dehydrogenase | bradi3g37240.1 | - | 2.3329 | - |
| **13** | 464 | amino acid metabolism.degradation.aromatic aa.tyrosine | bradi3g07130.1 | - | -2.49039 | - |
| **13** | 572 | amino acid metabolism.degradation.aromatic aa.tyrosine | bradi1g03130.1 | - | D | - |
| **13** | 192 | amino acid metabolism.synthesis.serine-glycine-cysteine group.cysteine.OASTL | bradi4g01130.2 | - | -6.17376 | - |
| **13** | 756 | amino acid metabolism.degradation.aspartate family.threonine | bradi5g03830.1 | - | - | 2.692307 |
| **12** | 586 | Nitrite Reductase 1 | bradi3g57990.1 | -2.73857 | -1.81951 | - |
| **12** | 41 | Glutamine synthetase | bradi5g24550.2 | -1.6112 | -1.53801 | - |
| **13** | 249 | Aspartate Aminotransferase 5 | bradi3g54200.1 | 1.576329 | 5.237107 | - |
| **13** | 661 | Aspartate aminotransferase | bradi2g50500.1 | -4.93876 | 2.16009 | - |
| **13** | 162 | L-alanine:2-oxoglutarate aminotransferase | bradi1g59220.2 | 1.891376 | 1.508573 | - |
| **13** | 762 | ornithine carbamoyltransferase | bradi3g52727.1 | new | 1.678402 | - |
| **13** | 890 | aspartate-semialdehyde dehydrogenase | bradi1g07910.1 | new | 4.758007 | - |
| **13** | 171 | Cysteine synthase | bradi2g53170.1 | -3.37442 | -11.1597 | - |
| **13** | 486 | 1-pyrroline-5-carboxylate dehydrogenase | bradi2g18550.1 | 2.44119 | 1.519773 | - |
| **13** | 525 | Pyrroline-5-carboxylate reductase | bradi2g60730.1 | 3.103217 | 1.976995 | - |
| **13** | 49 | Glycine cleavage system P protein | bradi2g48010.1 | 2.480919 | 2.303416 | - |
| **13** | 95 | Aminomethyltransferase | bradi5g22290.1 | 3.044691 | 1.922112 | - |
| **13** | 804 | amino acid metabolism.synthesis.central amino acid metabolism.GABA.SSADH | bradi3g05490.1 | - | 3.056931 | 1.78696 |
| **13** | 685 | 2-isopropylmalate synthase | bradi4g43130.1 | D | 1.926745 | 2.033443 |
| **Secondary metabolism (16)** | | | | | | |
| **16** | 793 | solanesyl diphosphate synthase 1 | bradi2g15307.1  3.354378 | | - | - |
| **16** | 706 | 1-deoxy-D-xylulose-5-phosphate reductoisomerase | bradi2g00650.1 | 2.569124 | - | - |
| **16** | 766 | / 4-hydroxy-3-methylbut-2-en-1-yl diphosphate synthase | bradi3g48080.1 | new | - | - |
| **16** | 496 | violaxanthin de-epoxidase | bradi5g07390.1 | -2.43078 |  | - |
| **16** | 225 | secondary metabolism.isoprenoids.non-mevalonate pathway | bradi3g59430.1 | - | 1.561997 | - |
| **16** | 729 | secondary metabolism.sulfur-containing.misc.alliinase | bradi2g48267.1 | - | 1.542854 | - |
| **16** | 581 | secondary metabolism.flavonoids.dihydroflavonols.dihydroflavonol 4-reductase | bradi1g35742.1 | - | -6.70729 | - |
| **16** | 806 | secondary metabolism.isoprenoids.mevalonate pathway.isopentenyl pyrophosphate:dimethyllallyl pyrophosphate isomerase | bradi1g25350.1 | - | D | - |
| **16** | 779 | 4-(cytidine 5'-diphospho)-2-C-methyl-D-erythritol kinase | bradi2g52610.1 | 1.926806 | new | - |
| **16** | 238 | S-adenosylmethionine-dependent methyltransferase | bradi4g01640.1 | 1.616621 | 1.773624 | - |
| **16** | 842 | isoflavone reductase | bradi4g05455.1 | D | D | - |
| **16** | 785 | homogentisate farnesyltransferase | bradi2g18210.1 | 2.297475 | 4.816604 | -5.89711 |
| **Hormone metabolism (17)** | | | | | | |
| **17** | 665 | AILP1 | bradi4g42890.1 | -2.81035 | - | - |
| **17** | 862 | unknown protein | bradi1g08930.1 | D | - | - |
| **17** | 504 | hormone metabolism.auxin.induced-regulated-responsive-activated | bradi2g43690.1 | - | 4.533726 | - |
| **17** | 515 | hormone metabolism.auxin.induced-regulated-responsive-activated | bradi3g48197.1 | - | 2.635557 | - |
| **17** | 655 | hormone metabolism.ethylene.synthesis-degradation | bradi3g57520.1 | - | 3.589018 | - |
| **17** | 546 | NCED4 | bradi3g52680.1 | 3.840365 | 2.37659 | - |
| **17** | 214 | NCED1, 9-cis-epoxycarotenoid dioxygenase | bradi4g00335.1 | 1.918931 | 1.518522 |  |
| **Stress (20)** | | | | | | |
| **20** | 124 | oxalate oxidase | bradi3g37670.1 | 2.26757 | - | - |
| **20** | 736 | stress.biotic | bradi4g05440.1 | - | 1.621153 | - |
| **20** | 62 | stress.abiotic.heat | bradi1g66590.1 | - | 1.517688 | - |
| **20** | 129 | stress.abiotic.heat | bradi3g39630.1 | - | 1.75309 | - |
| **20** | 255 | stress.abiotic.heat | bradi4g04220.1 | - | 1.553727 | - |
| **20** | 426 | stress.abiotic.heat | bradi4g32941.1 | - | 1.611915 | - |
| **20** | 451 | stress.abiotic.heat | bradi2g33682.1 | - | 1.780284 | - |
| **20** | 153 | stress.abiotic.drought/salt | bradi2g07940.1 | - | -2.52085 | - |
| **20** | 308 | stress.abiotic.heat | bradi1g66470.1 | - | - | 2.265283 |
| **20** | 523 | stress.abiotic.heat | bradi4g41057.1 | - | - | 2.084851 |
| **20** | 907 | DNAJ heat shock protein | bradi3g54550.1 | new | 1.784582 | - |
| **20** | 739 | ERD4 \| ERD4 (early-responsive to dehydration 4) | bradi1g58380.1 | D | 5.264365 | - |
| **20** | 550 | germin-like protein | bradi1g11920.1 | -16.9363 | -23.4745 | - |
| **20** | 822 | GL22 | bradi2g11050.1 | -5.37449 | -5.59635 | - |
| **20** | 472 | protein Leucine Rich Repeat family | bradi1g56690.1 | 3.975671 | 53.73592 | -2.60115 |
| **20** | 448 | universal stress protein (USP) | bradi2g34910.1 | 2.368555 | 3.452202 | -3.44858 |
| **Redox (21)** | | | | | | |
| **21** | 137 | Thioredoxin M-type | bradi4g40800.1 | 2.249759 | - | - |
| **21** | 276 | CB5-B (Cytochrome B5 ISOFORM B) | bradi2g62010.1 | -2.4168 | - | - |
| **21** | 358 | MSBP1 (membrane steroid binding protein 1) | bradi3g30180.1 | 4.006622 | - | - |
| **21** | 149 | L-ascorbate peroxidase | bradi1g16510.1 | 2.06315 | - | - |
| **21** | 240 | Glutathione peroxidase | bradi1g47140.1 | 1.716299 | - | - |
| **21** | 509 | glutathione-disulfide reductase | bradi3g55030.2 | 1.528786 | - | - |
| **21** | 84 | Catalase | bradi1g76330.2 | 1.507336 | - | - |
| **21** | 328 | NADH-cytochrome b5 reductase | bradi2g04610.1 | -2.62146 | - | - |
| **21** | 370 | redox.thioredoxin | bradi5g26060.1 | - | -4.44172 | - |
| **21** | 356 | Glutathione S-Transferase | bradi1g06450.1 | 2.34 | 3.9 | 4.8 |
| **21** | 145 | monodehydroascorbate reductase | bradi4g38430.1 | - | 2.52089 | - |
| **21** | 277 | L-ascorbate peroxidase | bradi1g45010.1 | - | 4.06015 | - |
| **21** | 391 | redox.ascorbate and glutathione.glutathione | bradi2g34770.1 | - | 2.485854 | - |
| **21** | 59 | redox.peroxiredoxin | bradi1g46620.1 | - | 2.74917 | - |
| **21** | 387 | redox.peroxiredoxin | bradi2g46380.2 | - | new | - |
| **21** | 143 | Catalase 3 | bradi3g01470.1 | - | 2.22378 | - |
| **21** | 119 | CHLOROPLASTIC DROUGHT-INDUCED STRESS PROTEIN OF 32 KD) | bradi1g28090.1 | 2.77205 | 1.783703 | - |
| **21** | 453 | disulfide isomerase | bradi2g35020.1 | -2.7281 | 1.727304 | - |
| **21** | 698 | Thioredoxin M-type | bradi5g16550.1 | 1.858813 | 2.39017 | - |
| **21** | 274 | Dehydroascorbate Reductase 2 | bradi2g37480.1 | 2.2426 | 2.164143 | - |
| **21** | 425 | ascorbate peroxidase 1 | bradi1g65820.2 | new | new | - |
| **21** | 802 | Monodehydroascorbate reductase | bradi3g17120.1 | new | new | - |
| **21** | 821 | Ascorbate Peroxidase 3 | bradi5g03640.1 | 5.184192 | 8.10606 | - |
| **21** | 125 | Dihydrolipoyl dehydrogenase | bradi2g12310.1 | 1.797906 | 2.426139 | - |
| **21** | 673 | heme-binding family protein | bradi1g36860.1 | new | new | - |
| **21** | 692 | glutathione disulfide oxidoreductase | bradi1g01570.1 | 1.937827 | 1.753645 |  |
| **21** | 61 | peroxiredoxin type 2 | bradi3g06750.1 | 2.370917 | 2.827772 | - |
| **21** | 522 | peroxiredoxin type 2 | bradi1g35660.1 | 13.22448 | new | - |
| **21** | 101 | superoxide dismutase | bradi3g43070.1 | 5.59261 | 4.28992 | - |
| **21** | 195 | Superoxide dismutase [Cu-Zn] | bradi1g18340.2 | 5.14377 | - | 1.629304 |
| **21** | 557 | Thioredoxin reductase | bradi1g19140.1 | 2.270563 | 2.715588 | D |
| **DNA/RNA (27, 28)** | | | | | | |
| **27 (RNA)** | 650 | LIL3:1; transcription factor | bradi3g02570.1 | -2.93284 | - | - |
| **27** | 832 | HMG1/2-like protein | bradi1g29730.4 | -7.3825 | - | - |
| **27** | 7 | pentatricopeptide (PPR) repeat-containing protein | bradi3g16227.1 | 2.202766 | - | - |
| **27** | 476 | DNA-binding protein | bradi5g16720.1 | -11.047 | - | - |
| **27** | 848 | Nascent polypeptide-associated complex subunit beta | bradi1g77957.1 | -16.4749 | - | - |
| **27** | 194 | CSP41A (CHLOROPLAST STEM-LOOP BINDING PROTEIN OF 41 KDA) | bradi1g54030.1 | 1.586148 | - | - |
| **27** | 722 | RNA binding | bradi2g36710.1 | -2.63417 | - | - |
| **27** | 870 | heterogeneous nuclear ribonucleoprotein | bradi3g28430.1 | -2.55125 | - | - |
| **28 (DNA)** | 75 | histone H3.2 | bradi3g45290.1 | -3.16184 | - | - |
| **28** | 239 | H2B | bradi2g27760.1 | -5.37509 | - | - |
| **28** | 414 | Histone H2A | bradi2g37327.1 | -7.82609 | - | - |
| **27** | 747 | RNA.processing.RNA helicase | bradi2g40800.1 | - | 1.959845 | - |
| **27** | 378 | RNA.processing.ribonucleases | bradi1g26277.1 | - | -2.08014 | - |
| **27** | 695 | RNA.regulation of transcription.MADS box transcription factor family | bradi4g11097.1 | - | 2.447552 | - |
| **27** | 902 | RNA.regulation of transcription.putative transcription regulator | bradi2g01860.1 | - | new | - |
| **28** | 886 | DNA.synthesis/chromatin structure.histone | bradi2g00530.1 | - | 5.370516 | - |
| **28** | 755 | DNA.unspecified | bradi1g10340.2 | - | -13.9158 | - |
| **27** | 770 | RNA.processing | bradi4g08430.1 | - | - | 3.224885 |
| **27** | 910 | RNA.processing.splicing | bradi4g09940.1 | - | - | 1.657794 |
| **27** | 110 | RNA.RNA binding | bradi4g38260.1 | - | - | -3.90413 |
| **27** | 120 | EIF4A1 (EUKARYOTIC TRANSLATION INITIATION FACTOR 4A1) | bradi1g34170.3 | 1.670132 | 1.880454 | - |
| **27** | 833 | NF-YC4 (NUCLEAR FACTOR Y, SUBUNIT C4) | bradi1g67980.1 | -2.55968 | D | - |
| **27** | 271 | PTAC4 (PLASTID TRANSCRIPTIONALLY ACTIVE4) | bradi1g18320.1 | -3.70652 | -3.68388 | - |
| **27** | 726 | chloroplast nucleoid DNA-binding protein | bradi3g56660.1 | -5.02725 | -2.09847 | - |
| **28** | 490 | NAP1;1 (NUCLEOSOME ASSEMBLY PROTEIN1;1) | bradi2g18330.1 | -4.07571 | -2.15132 | - |
| **28** | 20 | Histone H2B | bradi3g54520.1 | -3.59531 | -2.613 | - |
| **28** | 21 | Histone H4 | bradi4g37340.1 | -5.4847 | -2.28601 | - |
| **28** | 68 | Histone H2A | bradi2g62450.1 | -4.73965 | -3.63593 | - |
| **28** | 89 | Histone H2A | bradi4g06010.1 | -5.76761 | -2.48098 | - |
| **28** | 107 | Histone H2A | bradi1g66370.1 | -5.7503 | -3.35126 | - |
| **28** | 512 | Histone H2A | bradi1g09060.1 | -9.48572 | -2.24176 | - |
| **28** | 520 | no original description | bradi1g05330.1 | -8.90243 | -6.05896 | - |
| **28** | 748 | Histone H2A | bradi1g25400.1 | -13.9334 | -2.97934 | - |
| **27** | 570 | Nascent polypeptide-associated complex subunit beta | bradi3g29087.1 | -2.89578 | - | -2.27878 |
| **27** | 787 | CP33; RNA binding | bradi1g57190.1 | -2.17385 | -5.77674 | 3.386512 |
| **Protein (29)** |  |  |  |  |  |  |
| **29** | 684 | Alanine--tRNA ligase | bradi3g22760.1 | 2.503357 | - | - |
| **29** | 296 | Ribosomal protein S7 | bradi1g05710.1 | -4.86417 | - | - |
| **29** | 151 | ribosomal protein L1 family protein | bradi2g26940.1 | -3.82777 | - | - |
| **29** | 404 | a chloroplast ribosomal protein L14 | bradi3g27300.1 | -2.36443 | - | - |
| **29** | 297 | 50S ribosomal protein L24, chloroplast (CL24) | bradi1g33030.1 | -3.92941 | - | - |
| **29** | 701 | 50S ribosomal protein L33, chloroplastic | bradi1g05754.1 | -3.71952 | - | - |
| **29** | 224 | 40S ribosomal protein S10 (RPS10A) | bradi2g61560.1 | -2.98679 | - | - |
| **29** | 421 | 40S ribosomal protein S16 (RPS16A) | bradi4g26140.1 | -2.92162 | - | - |
| **29** | 709 | RPS18C (S18 RIBOSOMAL PROTEIN) | bradi1g05670.1 | -2.97084 | - | - |
| **29** | 329 | 40S ribosomal protein S19 (RPS19B) | bradi3g56527.1 | -3.18766 | - | - |
| **29** | 411 | 40S ribosomal protein S2 (RPS2D) | bradi1g04660.1 | -3.93174 | - | - |
| **29** | 459 | structural constituent of ribosome | bradi1g62690.2 | -3.13212 | - | - |
| **29** | 284 | 40S ribosomal protein S23 (RPS23B) | bradi2g54210.1 | -3.60878 | - | - |
| **29** | 365 | 40S ribosomal protein S9 (RPS9C) | bradi4g07120.1 | -3.9236 | - | - |
| **29** | 449 | 60S ribosomal protein L10 (RPL10C) | bradi2g34440.6 | -6.43818 | - | - |
| **29** | 197 | 60S ribosomal protein L12 (RPL12A) | bradi5g20330.1 | -4.22564 | - | - |
| **29** | 350 | 60S ribosomal protein L13 | bradi3g16170.3 | -4.4048 | - | - |
| **29** | 121 | 60S ribosomal protein L4/L1 (RPL4A) | bradi1g05510.1 | -2.68713 | - | - |
| **29** | 662 | Ribosomal protein L19 | bradi1g63010.2 | -3.24715 | - | - |
| **29** | 483 | 60S ribosomal protein L26 (RPL26B) | bradi2g06250.1 | -3.5298 | - | - |
| **29** | 307 | 60S ribosomal protein L27 | bradi1g30210.1 | -3.66935 | - | - |
| **29** | 657 | 60S ribosomal protein L32 (RPL32A) | bradi4g34340.2 | -6.85362 | - | - |
| **29** | 254 | 60S ribosomal protein L34 (RPL34A) | bradi3g16620.1 | -4.48485 | - | - |
| **29** | 369 | 60S ribosomal protein L36 | bradi2g54600.2 | -8.85022 | - | - |
| **29** | 229 | RPL5A | bradi5g09500.1 | -2.16748 | - | - |
| **29** | 519 | Ribosomal protein | bradi4g38510.1 | -5.20823 | - | - |
| **29** | 393 | 60S ribosomal protein L13A (RPL13aD) | bradi1g08260.1 | -2.10774 | - | - |
| **29** | 330 | 60S ribosomal protein L17 (RPL17A) | bradi4g08667.1 | -4.51226 | - | - |
| **29** | 285 | 60S ribosomal protein L21 (RPL21C) | bradi1g75620.1 | -2.70658 | - | - |
| **29** | 534 | 60S ribosomal protein L35a (RPL35aC) | bradi4g16690.3 | -4.78254 | - | - |
| **29** | 237 | 60S ribosomal protein L7A (RPL7aA) | bradi4g34750.1 | -2.53339 | - | - |
| **29** | 232 | 60S ribosomal protein L6 | bradi3g08900.1 | -4.30842 | - | - |
| **29** | 372 | 60S ribosomal protein L8 (RPL8C) | bradi4g04120.2 | -2.6227 | - | - |
| **29** | 56 | Elongation factor Tu | bradi3g47690.1 | 1.649265 | - | - |
| **29** | 138 | Elongation factor G, chloroplastic | bradi5g16980.1 | 2.609627 | - | - |
| **29** | 415 | elongation factor 1B alpha-subunit 2 (eEF1Balpha2) | bradi1g18710.1 | -2.2071 | - | - |
| **29** | 351 | mitochondrial processing peptidase beta subunit | bradi1g70180.1 | 2.052591 | - | - |
| **29** | 178 | translocon at the inner envelope membrane of chloroplasts | bradi3g29750.1 | 2.210724 | - | - |
| **29** | 441 | TIC55 (TRANSLOCON AT THE INNER ENVELOPE MEMBRANE OF CHLOROPLASTS 55) | bradi3g53860.1 | 2.799066 | - | - |
| **29** | 740 | protein transport protein SEC61 gamma subunit | bradi1g31180.1 | -2.03689 | - | - |
| **29** | 189 | protein serine/threonine kinase | bradi1g08287.1 | 1.66162 | - | - |
| **29** | 473 | ATP-dependent Clp protease proteolytic subunit | bradi1g05750.1 | 4.399978 | - | - |
| **29** | 435 | aspartyl protease family protein | bradi2g16160.1 | -2.71733 | - | - |
| **29** | 605 | serine carboxypeptidase S28 family protein | bradi3g30640.1 | -4.8784 | - | - |
| **29** | 190 | CPN20 (CHAPERONIN 20) | bradi4g31220.1 | 2.90669 | - | - |
| **29** | 377 | GrpE protein homolog | bradi3g48540.1 | -2.62711 | - | - |
| **29** | 446 | CPN20 (CHAPERONIN 20) | bradi1g46596.1 | 1.552573 | - | - |
| **29** | 733 | GrpE protein homolog | bradi4g27270.1 | D | - | - |
| **29** | 812 | PDF6 (PREFOLDIN 6) | bradi3g09300.1 | -7.35741 | - | - |
| **29** | 111 | photosystem II stability/assembly factor | bradi1g29500.1 | 1.969052 | - | - |
| **29** | 691 | photosystem I assembly and stability | bradi2g13078.1 | 2.232597 | - | - |
| **29** | 717 | Eukaryotic translation initiation factor 5A | bradi1g59110.1 | -2.2801 | - | - |
| **29** | 352 | Proteasome subunit alpha type | bradi3g49610.2 | -2.16804 | - | - |
| **29** | 210 | RD21 (responsive to dehydration 21) | bradi5g24027.1 | -2.59389 | - | - |
| **29** | 727 | cysteine-type peptidase | bradi4g31440.3 | -7.67877 | - | - |
| **29** | 889 | aspartyl protease family protein | bradi4g36720.2 | new | - | - |
| **29** | 338 | protein.synthesis.ribosomal protein.prokaryotic.chloroplast.30S subunit.S18 | bradi1g05752.1 | - | -2.23136 | - |
| **29** | 230 | protein.synthesis.ribosomal protein.eukaryotic.40S subunit.S3 | bradi3g16150.1 | - | 2.411518 | - |
| **29** | 629 | protein.synthesis.ribosomal protein.eukaryotic.40S subunit.S24 | bradi3g08680.1 | - | -2.39151 | - |
| **29** | 852 | protein.synthesis.ribosomal protein.eukaryotic.40S subunit.S27 | bradi3g43475.1 | - | 1.504212 | - |
| **29** | 531 | protein.synthesis.ribosomal protein.eukaryotic.40S subunit.S28 | bradi4g13910.1 | - | 3.582534 | - |
| **29** | 193 | protein.synthesis.ribosomal protein.eukaryotic.40S subunit.SA | bradi1g23780.1 | - | 1.68777 | - |
| **29** | 182 | protein.synthesis.ribosomal protein.eukaryotic.40S subunit.S4 | bradi2g35620.1 | - | 3.034154 | - |
| **29** | 544 | protein.synthesis.ribosomal protein.eukaryotic.40S subunit.S6 | bradi1g21320.2 | - | 2.8355 | - |
| **29** | 220 | protein.synthesis.ribosomal protein.eukaryotic.60S subunit.L3 | bradi4g41680.1 | - | 1.704351 | - |
| **29** | 510 | protein.synthesis.ribosomal protein.eukaryotic.60S subunit.P1 | bradi3g13750.1 | - | new | - |
| **29** | 205 | protein.synthesis.ribosomal protein.eukaryotic.60S subunit.L9 | bradi3g00640.1 | - | 3.250718 | - |
| **29** | 567 | protein.synthesis.initiation | bradi3g53970.2 | - | -9.61453 | - |
| **29** | 81 | protein.synthesis.elongation | bradi1g06860.8 | - | 4.58633 | - |
| **29** | 223 | protein.synthesis.elongation | bradi1g37490.1 | - | 2.980046 | - |
| **29** | 462 | protein.targeting.mitochondria | bradi2g05710.1 | - | 1.776368 | - |
| **29** | 690 | protein.targeting.chloroplast | bradi2g03350.1 | - | 3.249885 | - |
| **29** | 825 | protein.targeting.chloroplast | bradi2g35587.1 | - | -2.37673 | - |
| **29** | 899 | protein.targeting.chloroplast | bradi1g66820.1 | - | new | - |
| **29** | 635 | protein.degradation | bradi3g60550.1 | - | 2.113161 | - |
| **29** | 880 | protein.degradation | bradi3g35350.1 | - | new | - |
| **29** | 488 | protein.degradation.ubiquitin.proteasom | bradi5g24960.1 | - | -2.50597 | - |
| **29** | 551 | protein.degradation.ubiquitin.proteasom | bradi2g33390.2 | - | 3.12345 | - |
| **29** | 588 | protein.degradation.ubiquitin.proteasom | bradi3g07370.4 | - | 1.920749 | - |
| **29** | 631 | protein.degradation.ubiquitin.proteasom | bradi5g13800.1 | - | 2.800021 | - |
| **29** | 688 | protein.degradation.ubiquitin.proteasom | bradi4g35156.2 | - | -4.48593 | - |
| **29** | 705 | protein.degradation.ubiquitin.proteasom | bradi3g03720.1 | - | 3.223939 | - |
| **29** | 751 | protein.degradation.ubiquitin.proteasom | bradi4g34620.1 | - | new | - |
| **29** | 794 | protein.degradation.ubiquitin.proteasom | bradi1g49140.1 | - | 1.612003 | - |
| **29** | 140 | protein.degradation.serine protease | bradi4g39880.1 | - | 1.828929 | - |
| **29** | 43 | protein.folding | bradi1g50270.1 | - | 2.110535 | - |
| **29** | 116 | protein.folding | bradi1g00730.1 | - | 1.5706 | - |
| **29** | 260 | protein.folding | bradi3g41800.1 | - | -2.35206 | - |
| **29** | 312 | protein.folding | bradi1g75520.1 | - | 3.384791 | - |
| **29** | 334 | protein.folding | bradi3g00480.2 | - | 4.186734 | - |
| **29** | 392 | protein.folding | bradi1g54970.1 | - | -2.12366 | - |
| **29** | 558 | protein.folding | bradi5g02990.2 | - | -3.7227 | - |
| **29** | 595 | protein.assembly and cofactor ligation | bradi1g63380.1 | - | -2.01256 | - |
| **29** | 261 | protein.synthesis.ribosomal protein.prokaryotic.chloroplast.30S subunit.S4 | bradi2g13104.1 | - | - | -2.03547 |
| **29** | 98 | protein.synthesis.ribosomal protein.prokaryotic.chloroplast.50S subunit.L12 | bradi2g45730.1 | - | - | -2.92665 |
| **29** | 253 | protein.synthesis.ribosomal protein.prokaryotic.chloroplast.50S subunit.L15 | bradi1g69650.1 | - | - | -2.46165 |
| **29** | 810 | protein.synthesis.ribosomal protein.eukaryotic.40S subunit.S11 | bradi5g21630.1 | - | - | 1.93791 |
| **29** | 790 | protein.synthesis.ribosomal protein.unknown.small subunit.S19 | bradi1g71472.1 | - | - | -2.20875 |
| **29** | 247 | protein.synthesis.elongation | bradi3g05950.1 | - | - | -2.51345 |
| **29** | 675 | protein.degradation.ubiquitin.proteasom | bradi2g27390.1 | - | - | D |
| **29** | 442 | tRNA synthetase class II | bradi4g07854.1 | 1.640946 | -3.03675 | - |
| **29** | 769 | aminoacyl-tRNA ligase | bradi1g56640.2 | 2.144624 | new | - |
| **29** | 827 | aminoacyl-tRNA ligase | bradi1g56890.1 | 3.304393 | 3.537549 | - |
| **29** | 868 | 30S ribosomal protein S15, chloroplastic | bradi3g27272.1 | -6.30596 | D | - |
| **29** | 768 | chloroplast 30S ribosomal protein S20 | bradi2g46520.1 | -15.0447 | -4.30738 | - |
| **29** | 471 | 30S ribosomal protein | bradi1g00980.2 | -2.1717 | -2.1138 | - |
| **29** | 492 | emb1473 (embryo defective 1473); structural constituent of ribosome | bradi2g49850.1 | -3.55138 | -8.66322 | - |
| **29** | 487 | ribosomal protein L17 family protein | bradi1g04130.2 | -7.7731 | -2.17765 | - |
| **29** | 355 | chloroplast ribosomal protein L23 | bradi5g05656.1 | -3.3916 | -3.37007 | - |
| **29** | 256 | RIBOSOMAL PROTEIN LARGE SUBUNIT 27 | bradi2g59210.1 | -3.40664 | -3.21803 | - |
| **29** | 659 | 50S ribosomal protein L28 | bradi2g40040.1 | -9.13006 | -2.23672 | - |
| **29** | 416 | ribosomal protein L29 | bradi3g58780.1 | -8.04118 | -26.5967 | - |
| **29** | 147 | RPL4; poly(U) binding / structural constituent of ribosome | bradi1g67170.1 | -2.49404 | -2.2007 | - |
| **29** | 612 | structural constituent of ribosome | bradi3g13810.1 | -6.37474 | -2.14668 | - |
| **29** | 258 | 40S ribosomal protein S14 | bradi3g45000.1 | -2.12596 | 2.432429 | - |
| **29** | 851 | 40S ribosomal protein S25 | bradi4g38580.1 | -8.99739 | D | - |
| **29** | 514 | 40S ribosomal protein S26 | bradi1g04070.1 | -2.72279 | 1.592593 | - |
| **29** | 477 | 40S ribosomal protein S15A | bradi2g54450.1 | -5.37765 | -2.63185 | - |
| **29** | 400 | 40S ribosomal protein S8 | bradi3g43560.1 | -3.45177 | 1.915357 | - |
| **29** | 246 | RPL16A; structural constituent of ribosome | bradi3g09030.1 | 4.440864 | 1.6107 | - |
| **29** | 478 | 60S ribosomal protein L4/L1 (RPL4A) | bradi1g55510.1 | -2.29468 | 2.129898 | - |
| **29** | 627 | 60S ribosomal protein L18 | bradi1g17970.1 | -4.669 | new | - |
| **29** | 272 | 60S ribosomal protein L18a | bradi2g50020.1 | -3.62378 | -2.4449 | - |
| **29** | 304 | RPL23A | bradi5g14750.2 | -10.0242 | -2.31178 | - |
| **29** | 590 | structural constituent of ribosome | bradi1g21630.1 | -4.38091 | -7.97999 | - |
| **29** | 781 | 60S ribosomal protein L37a (RPL37aB) | bradi2g46580.1 | -2.96767 | D | - |
| **29** | 216 | 60S ribosomal protein L7 (RPL7D) | bradi5g20900.1 | -3.74588 | -2.13615 | - |
| **29** | 201 | 60S acidic ribosomal protein P0 (RPP0A) | bradi3g14340.1 | -2.00925 | 1.82004 | - |
| **29** | 616 | protein ribosome-like protein | bradi3g06530.1 | -6.89611 | -3.46813 | - |
| **29** | 288 | nascent polypeptide associated complex alpha chain protein | bradi2g27190.1 | -4.24523 | -2.23281 |  |
| **29** | 460 | NACA2 (NASCENT POLYPEPTIDE-ASSOCIATED COMPLEX SUBUNIT ALPHA-LIKE PROTEIN 2) | bradi1g77100.1 | -2.37399 | -2.24275 | - |
| **29** | 713 | translation elongation factor | bradi2g45070.1 | new | 4.073928 | - |
| **29** | 275 | RRF (RIBOSOME RECYCLING FACTOR, CHLOROPLAST PRECURSOR) | bradi1g24110.2 | -5.92894 | -2.18245 | - |
| **29** | 131 | THF1 | bradi1g24860.3 | -3.33779 | -8.94675 | - |
| **29** | 856 | NTF2A (NUCLEAR TRANSPORT FACTOR 2A) | bradi3g41310.1 | -5.60727 | D | - |
| **29** | 860 | nuclear transport factor 2 (NTF2) | bradi5g07090.2 | D | -2.79283 | - |
| **29** | 658 | SCY1 (SecY Homolog 1); P-P-bond-hydrolysis-driven protein transmembrane transporter | bradi3g19100.1 | 1.795177 | 2.785402 | - |
| **29** | 694 | Protein translocase subunit SecA | bradi2g12067.1 | 2.259303 | 2.46328 | - |
| **29** | 380 | ARF1A1C | bradi1g53867.1 | -2.3087 | -2.01653 | - |
| **29** | 809 | Protein transport protein Sec61 subunit beta | bradi4g45150.1 | -6.69458 | -3.41711 | - |
| **29** | 805 | STN8 | bradi2g22210.1 | 2.783565 | new | - |
| **29** | 234 | cytosol aminopeptidase | bradi3g54020.1 | 1.96565 | 2.574428 | - |
| **29** | 323 | unknown protein | bradi3g47890.1 | -2.05635 | -2.57418 | - |
| **29** | 376 | serine-type endopeptidase | bradi3g56280.1 | 3.517865 | 4.450129 | - |
| **29** | 543 | acylaminoacyl-peptidase | bradi3g26780.4 | 1.53447 | 2.260805 | - |
| **29** | 577 | peptidase M1 | bradi3g12520.2 | 2.658947 | 1.59857 | - |
| **29** | 754 | Aminopeptidase | bradi3g08120.1 | new | new | - |
| **29** | 789 | ubiquitin activating enzyme | bradi1g65320.1 | 1.50735 | 7.08464 | - |
| **29** | 402 | aspartyl protease | bradi2g36862.1 | -2.19908 | -3.40828 | - |
| **29** | 580 | ubiquitin-protein ligase | bradi3g00340.1 | -2.68835 | D | - |
| **29** | 782 | aspartyl aminopeptidase | bradi4g39590.1 | new | D | - |
| **29** | 150 | ATPREP1 (PRESEQUENCE PROTEASE 1) | bradi3g58166.1 | 2.385411 | 2.217318 | - |
| **29** | 535 | membrane-associated zinc metalloprotease | bradi1g15560.1 | 4.128605 | 2.090935 | - |
| **29** | 562 | aminopeptidase | bradi2g28680.2 | -3.20313 | 1.624505 | - |
| **29** | 807 | protein.degradation.AAA type | bradi1g74922.1 | - | 2.626195 | - |
| **29** | 73 | CPN60A (CHAPERONIN-60ALPHA) | bradi5g02890.2 | 2.198269 | 3.109458 | - |
| **29** | 385 | ERD1 (EARLY RESPONSIVE TO DEHYDRATION 1) | bradi3g44640.1 | 2.002505 | 1.974712 | - |
| **29** | 468 | HSP60 (HEAT SHOCK PROTEIN 60) | bradi3g28070.1 | new | new | - |
| **29** | 563 | Peptidylprolyl isomerase | bradi1g27190.5 | 1.598661 | 2.284253 | - |
| **29** | 578 | mitochondrial import inner membrane translocase subunit Tim17 | bradi1g64590.1 | 8.141351 | 6.100464 | - |
| **29** | 608 | 50S ribosomal protein L35 | bradi0007s0021 | -4.79924 | - | -2.18939 |
| **29** | 801 | protein 50S ribosomal | bradi2g52710.1 | -12.6449 | - | 2.215075 |
| **29** | 772 | Small ubiquitin-related modifier | bradi2g58830.1 | -2.37951 | - | 1.882823 |
| **29** | 623 | Proteasome subunit alpha type | bradi4g13740.2 | -32.4703 | - | -2.20083 |
| **29** | 646 | Peptidylprolyl isomerase | bradi3g01630.1 | 1.505743 | - | -4.58092 |
| **29** | 395 | protein.synthesis.ribosomal protein.eukaryotic.40S subunit.S7 | bradi3g23310.1 | - | 1.610313 | -2.44371 |
| **29** | 571 | protein.synthesis.ribosomal protein.eukaryotic.60S subunit.L3 | bradi4g24610.1 | - | 2.850109 | D |
| **29** | 879 | protein.synthesis.elongation | bradi1g01580.1 | - | 2.100885 | new |
| **29** | 871 | 60S ribosomal protein L22-2 (RPL22B) | bradi1g62830.1 | -5.42229 | D | D |
| **29** | 865 | nascent polypeptide associated complex alpha chain protein | bradi1g62360.1 | D | D | new |
| **29** | 209 | TIC110 (TRANSLOCON AT THE INNER ENVELOPE MEMBRANE OF CHLOROPLASTS 110) | bradi3g29760.1 | 1.809457 | D | -2.52611 |
| **29** | 461 | ribosomal protein L18 family protein | bradi1g03220.1 | 2.762943 | -2.11487 | -4.62525 |
| **29** | 696 | 40S ribosomal protein S15 | bradi2g20390.1 | new | 1.587623 | -2.31671 |
| **29** | 367 | GrpE protein homolog | bradi5g10250.1 | 1.522559 | -5.0556 | -6.50321 |
| **29** | 493 | Peptidylprolyl isomerase | bradi3g59057.2 | 1.672863 | -3.3064 | -2.96201 |
| **Cell/signaling/development (30, 31, 33)** |  |  |  |  |  |  |
| **30** | 270 | NAD(P)-binding Rossmann-fold superfamily protein | bradi3g09010.2 | 2.642524 | - | - |
| **30** | 861 | leucine-rich repeat transmembrane protein kinase | bradi2g43110.1 | -3.75679 | - | - |
| **30** | 484 | GTP binding | bradi2g12510.1 | -2.16109 | - | - |
| **30** | 498 | Obg-like ATPase 1 | bradi1g06772.1 | 2.504512 | - | - |
| **30** | 440 | GTP-binding nuclear protein | bradi2g15730.1 | -3.86634 | - | - |
| **30** | 554 | Obg-like ATPase 1 | bradi3g17680.1 | 1.59451 | - | - |
| **30** | 305 | GF14b protein | bradi3g38640.2 | 2.160802 | - | - |
| **31** | 206 | plastid-lipid associated protein PAP | bradi4g14630.1 | -2.4425 | - | - |
| **31** | 302 | Annexin | bradi3g58830.1 | 1.683127 | - | - |
| **31** | 830 | Annexin | bradi4g29680.2 | -2.13562 | - | - |
| **31** | 432 | peptidyl-prolyl cis-trans isomerase | bradi1g24500.1 | D | - | - |
| **31** | 829 | Peptidylprolyl isomerase | bradi2g58640.1 | D | - | - |
| **33** | 670 | cupin family protein | bradi2g37470.1 | -9.3648 | - | - |
| **30** | 463 | signalling.light | bradi4g33940.1 | - | D | - |
| **30** | 231 | signalling.calcium | bradi2g57537.1 | - | -2.94629 | - |
| **30** | 831 | signalling.calcium | bradi1g68010.1 | - | -11.0203 | - |
| **30** | 343 | signalling.G-proteins | bradi2g41180.1 | - | 2.376508 | - |
| **30** | 344 | signalling.G-proteins | bradi2g19750.2 | - | 1.980871 | - |
| **30** | 914 | signalling.G-proteins | bradi5g13000.1 | - | 2.588728 | - |
| **30** | 410 | signalling.14-3-3 proteins | bradi4g16640.1 | - | 9.589595 | - |
| **31** | 96 | cell.organisation | bradi3g30710.2 | - | 1.806944 | - |
| **31** | 674 | cell.organisation | bradi2g39590.1 | - | 1.67949 | - |
| **31** | 470 | cell.cycle.peptidylprolyl isomerase | bradi2g11060.1 | - | -2.12336 | - |
| **31** | 664 | cell.cell death.plants | bradi1g75270.1 | - | 7.349729 | - |
| **33** | 850 | development.late embryogenesis abundant | bradi1g02090.2 | - | -3.61818 | - |
| **33** | 437 | development.unspecified | bradi3g14650.1 | - | -2.30059 | - |
| **33** | 702 | development.unspecified | bradi3g41430.1 | - | D | - |
| **33** | 874 | development.unspecified | bradi2g47940.1 | - | 3.735882 | - |
| **30** | 379 | CRT1 (CALRETICULIN 1) | bradi1g02940.2 | -3.38748 | -3.59889 | - |
| **30** | 405 | sodium/calcium exchanger family protein | bradi2g06830.1 | 1.989657 | 3.676624 | - |
| **30** | 869 | CAM5 (CALMODULIN 5) | bradi2g52940.1 | D | -2.65477 | - |
| **30** | 480 | GTP binding | bradi3g47240.1 | 1.881762 | 3.362543 | - |
| **30** | 573 | GTP binding | bradi1g03490.1 | -2.57212 | 4.664529 | - |
| **30** | 839 | GTP binding | bradi2g23120.1 | new | 1.805323 | - |
| **30** | 292 | 14-3-3-like protein | bradi5g12510.2 | -5.00887 | -2.75354 | - |
| **30** | 846 | RALFL33 (ralf-like 33) | bradi4g05500.1 | -18.3522 | D | - |
| **31** | 345 | Tubulin beta chain | bradi1g06800.1 | -2.89759 | 2.332958 | - |
| **31** | 778 | plastid-lipid associated protein PAP | bradi3g30740.2 | new | 1.568683 | - |
| **31** | 808 | Peptidylprolyl isomerase | bradi4g34370.1 | -3.41182 | -5.90996 | - |
| **31** | 888 | Clathrin heavy chain | bradi4g26877.1 | new | new | - |
| **30** | 419 | CAM5 (CALMODULIN 5) | bradi2g21460.1 | -6.92174 | - | 1.557196 |
| **Transport (34)** |  |  |  |  |  |  |
| **34** | 837 | (NON-INTRINSIC ABC PROTEIN 6) | bradi2g01870.1 | new | - | - |
| **34** | 828 | PIP2B (PLASMA MEMBRANE INTRINSIC PROTEIN 2); water channel | bradi5g15970.1 | new | - | - |
| **34** | 181 | antiporter/ triose-phosphate transmembrane transporter | bradi2g08340.1 | 1.643869 | - | - |
| **34** | 154 | transport.p- and v-ATPases | bradi5g24690.1 | - | 3.163213 | - |
| **34** | 104 | transport.p- and v-ATPases.H+-transporting two-sector ATPase | bradi1g31690.2 | - | 1.997175 | - |
| **34** | 652 | transport.ABC transporters and multidrug resistance systems | bradi2g01610.1 | - | -3.74029 | - |
| **34** | 134 | transport.Major Intrinsic Proteins.PIP | bradi1g28760.2 | - | 2.271713 | - |
| **34** | 167 | transport.Major Intrinsic Proteins.PIP | bradi3g56020.1 | - | 1.997584 | - |
| **34** | 669 | transport.calcium | bradi4g45030.1 | - | 2.553294 | - |
| **34** | 602 | transport.metabolite transporters at the mitochondrial membrane | bradi1g71410.2 | - | - | 2.005531 |
| **34** | 268 | transport.misc | bradi3g48590.1 | - | - | 1.762503 |
| **34** | 132 | transport.unspecified anions.arsenite-transporting ATPase | bradi3g59400.1 | - | - | -2.20085 |
| **34** | 540 | V-type proton ATPase subunit a | bradi1g67960.1 | 4.788455 | 3.025514 | - |
| **34** | 784 | V-type proton ATPase subunit G | bradi5g20650.1 | -12.6746 | 3.338263 | - |
| **34** | 389 | VHA-E3 (VACUOLAR H+-ATPASE SUBUNIT E ISOFORM 3) | bradi2g45580.1 | -4.43566 | 1.768395 | - |
| **34** | 575 | V-type proton ATPase subunit | bradi2g42100.1 | 4.2517 | 2.642825 | - |
| **34** | 613 | non-intrinsic ABC protein 8 | bradi1g28416.2 | new | 7.906712 | - |
| **34** | 438 | PIP2B (PLASMA MEMBRANE INTRINSIC PROTEIN 2); water channel | bradi3g49360.1 | new | 2.440507 | - |
| **34** | 530 | PIP1;4 (PLASMA MEMBRANE INTRINSIC PROTEIN 1;4); water channel | bradi5g18170.1 | 2.745458 | 3.03425 | - |
| **34** | 763 | TMT2 (TONOPLAST MONOSACCHARIDE TRANSPORTER2) | bradi3g32210.2 | 1.954733 | new | - |
| **34** | 909 | SUT4 (Sucrose Transporter 4) | bradi4g00320.1 | new | 5.386176 | - |
| **34** | 233 | (Pyrophosphate-energized inorganic pyrophosphatase) (H(+)-PPase) | bradi1g47767.1 | 2.894392 | 5.257463 | - |
| **34** | 841 | amino acid transporter family protein | bradi3g53740.1 | new | new | - |
| **34** | 813 | ADP,ATP carrier protein | bradi3g07830.1 | new | new | - |
| **34** | 863 | antiporter/ triose-phosphate transmembrane transporter | bradi4g27550.1 | D | 1.999915 | - |
| **34** | 217 | dicarboxylate/tricarboxylate carrier (DTC) | bradi2g32600.2 | 1.993031 | 1.864631 | - |
| **34** | 221 | mitochondrial phosphate transporter | bradi3g57890.1 | 1.713796 | 2.900163 | - |
| **34** | 474 | oxoglutarate:malate antiporter | bradi3g38580.1 | new | 2.261047 | - |
| **34** | 517 | TEMPERATURE-INDUCED LIPOCALIN | bradi3g36970.1 | 1.724205 | 5.162512 | - |
| **34** | 450 | transport.metabolite transporters at the mitochondrial membrane | bradi3g34077.1 | - | -2.06711 | -2.33428 |
