# Supplementary figures and images for: Comparative physiological and proteomic analysis indicates lower shock response to drought stress conditions in a self-pollinating perennial ryegrass
Source: PLoS One. 2020 Jun 18;15(6):e0234317. doi: 10.1371/journal.pone.0234317 (PMC7302502; doi:10.1371/journal.pone.0234317)

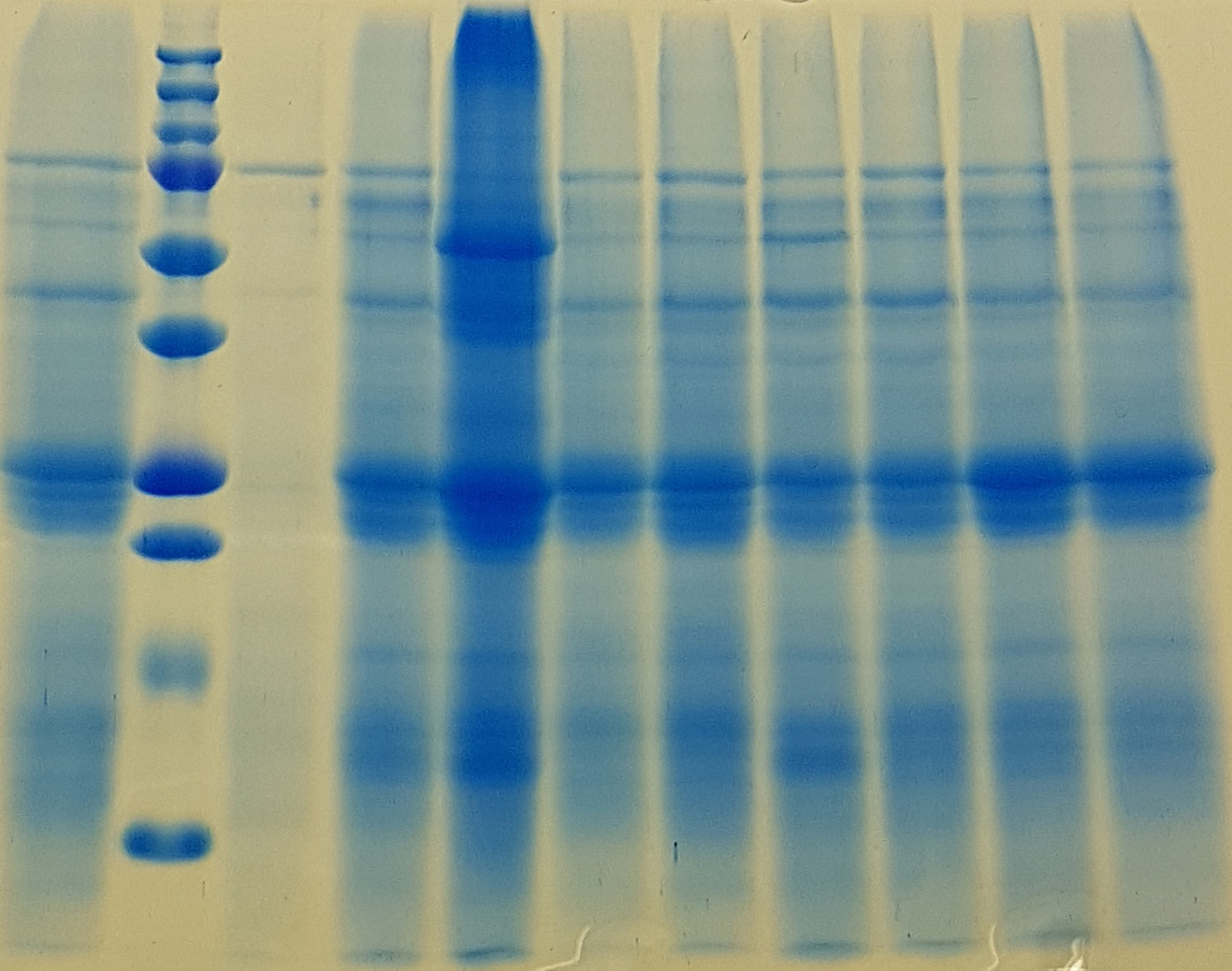

Supplement: S1 Fig — Left to right: Protein marker (lane 1), Control sample of Vigor genotype (lane 2,3), Drought sample of Vigor genotype (lane 4,5), Drought sample of Speedy genotype (lane 6), Control sample of Speedy genotype (lane 7), Control sample of S10 genotype (lane 8,9), Drought sample of S10 genotype (lane 10, 11). (TIF) [file pone.0234317.s002.tif]
